# A computational approach for identifying pathogenicity islands in prokaryotic genomes

**DOI:** 10.1186/1471-2105-6-184

**Published:** 2005-07-21

**Authors:** Sung Ho Yoon, Cheol-Goo Hur, Ho-Young Kang, Yeoun Hee Kim, Tae Kwang Oh, Jihyun F Kim

**Affiliations:** 1 Genome Research Center, Korea Research Institute of Bioscience and Biotechnology (KRIBB), 52 Oun-dong, Yuseong, Daejeon 305-333, Korea; 2 21C Frontier Microbial Genomics and Applications Center, KRIBB, 52 Oun-dong, Yuseong, Daejeon 305-333, Korea

## Abstract

**Background:**

Pathogenicity islands (PAIs), distinct genomic segments of pathogens encoding virulence factors, represent a subgroup of genomic islands (GIs) that have been acquired by horizontal gene transfer event. Up to now, computational approaches for identifying PAIs have been focused on the detection of genomic regions which only differ from the rest of the genome in their base composition and codon usage. These approaches often lead to the identification of genomic islands, rather than PAIs.

**Results:**

We present a computational method for detecting potential PAIs in complete prokaryotic genomes by combining sequence similarities and abnormalities in genomic composition. We first collected 207 GenBank accessions containing either part or all of the reported PAI loci. In sequenced genomes, strips of PAI-homologs were defined based on the proximity of the homologs of genes in the same PAI accession. An algorithm reminiscent of sequence-assembly procedure was then devised to merge overlapping or adjacent genomic strips into a large genomic region. Among the defined genomic regions, PAI-like regions were identified by the presence of homolog(s) of virulence genes. Also, GIs were postulated by calculating G+C content anomalies and codon usage bias. Of 148 prokaryotic genomes examined, 23 pathogenic and 6 non-pathogenic bacteria contained 77 candidate PAIs that partly or entirely overlap GIs.

**Conclusion:**

Supporting the validity of our method, included in the list of candidate PAIs were thirty four PAIs previously identified from genome sequencing papers. Furthermore, in some instances, our method was able to detect entire PAIs for those only partial sequences are available. Our method was proven to be an efficient method for demarcating the potential PAIs in our study. Also, the function(s) and origin(s) of a candidate PAI can be inferred by investigating the PAI queries comprising it. Identification and analysis of potential PAIs in prokaryotic genomes will broaden our knowledge on the structure and properties of PAIs and the evolution of bacterial pathogenesis.

## Background

PAIs are distinct genetic elements of pathogens encoding various virulence factors such as protein secretion systems, host invasion factors, iron uptake systems, and toxins [1,2]. PAIs are a subset of genomic islands which have been transferred by horizontal gene transfer (HGT) event and confer virulence upon the recipient. PAIs can be identified by features such as the presence of virulence genes, biased G+C content and codon usage, carriage of mobile sequence elements, and/or association with tRNA genes or repeated sequences at their boundaries [3].

Identification of PAIs is essential in understanding the development of disease and the evolution of bacterial pathogenesis [2]. As complete genome sequences rapidly accumulate, various *in silico *methods have been developed to detect HGT [4-7]. Most of the methods were based on the detection of genomic regions having atypical G+C content, patterns of codon usage bias, or dinucleotide anomaly. However, compositional approaches may generate many false positives due to other factors such as selection and mutation bias [8,9], and a lot of false negatives owing to adjustment of the transferred sequence in its composition by amelioration [10]. In fact, these methods detect different sets of ORFs as foreign origin when applied to the genome of *Escherichia coli *K-12 [11]. Thus, combining multiple lines of evidence can be beneficial to determine whether a gene or a group of genes has been acquired by HGT.

While studies on detecting horizontally transferred genes or GIs in genome sequences have been intensively carried out, little has been reported for PAIs. Considering that a PAI is a GI encoding virulence factors, compositional criteria such as G+C content and codon usage is not sufficient for identifying PAIs because genomic approaches can only lead to the identification of GIs [2]. In this work, we designed a computational method for identifying PAIs in sequenced genomes by combining a homology-based method and detection of abnormalities in genomic composition. To do this, we collected published PAI data and checked virulence genes on the PAI loci. We applied this approach to 148 prokaryotic genomes and identified 77 candidate PAIs. Detected regions contain virulence genes and relics of the HGT event.

## Results

### Genomic islands in bacterial genomes

As for the 157 chromosomes examined (Table 1S [see Additional file 1]), the length proportion of GIs to the chromosome averaged 10.1%. *Nanoarchaeum equitans*, the smallest genome of any sequenced microbes, contained the smallest proportion of GIs, which is only 2.9%. *Leptospira intrerrogans*, which is responsible for worldwide water-borne zoonosis leptospirosis, contained the largest, 34.7% for chromosome I and 32.2% for chromosome II. The genome of *L. interrogans *was reported to have the biggest number of proteins with structural similarity to eukaryal and archaeal proteins as compared to other bacteria [12]. In general, larger proportions of GIs in pathogens than those in related nonpathogenic species were observed, e.g., 15.7% for *Corynebacterium diphtheriae *versus 7.6% for *C. glutamicum*, 12.3% for *E. coli *CFT073 versus 8.9% for *E. coli *K-12.

### PAI-like regions

**Figure 1:**
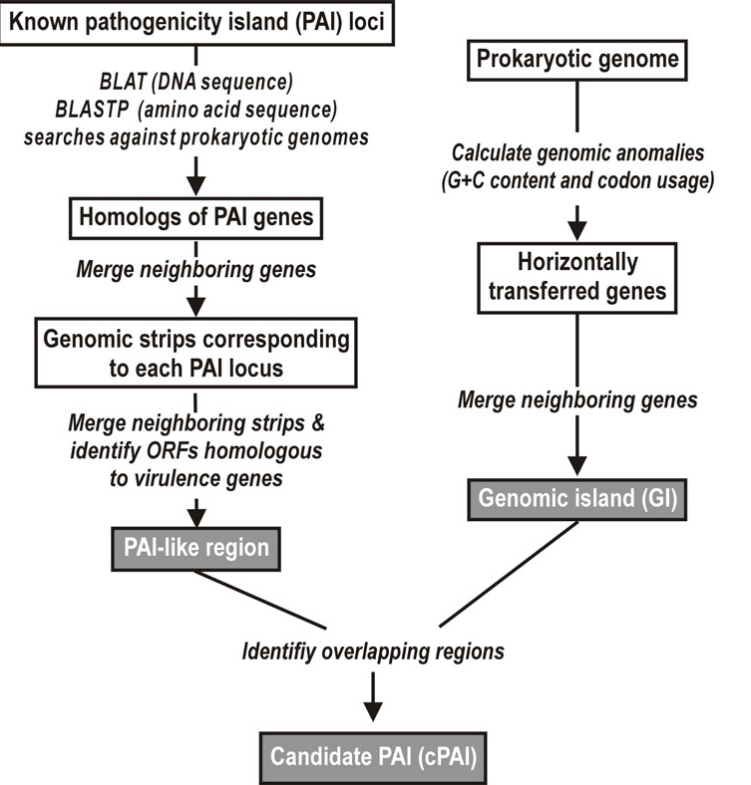
Flow chart of the algorithm.

**Figure 2:**
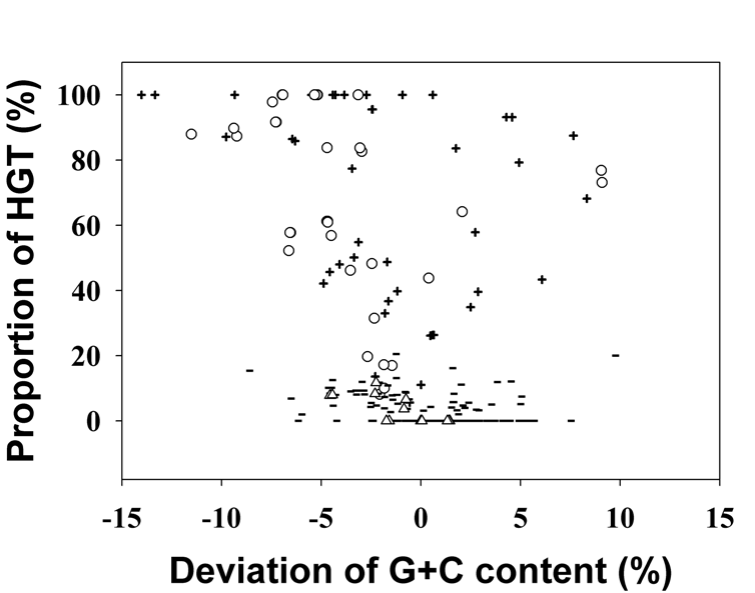
**Projection of PAI-like regions in their G+C contents and length-proportion of horizontally transferred genes. **Projection of PAI-like regions which overlap genomic islands (cPAI) and those which do not overlap genomic islands (nPAI) in their G+C contents (*X *axis) and length-proportion of horizontally transferred genes (*Y *axis). Each symbols denotes follows; cPAI (plus sign), nPAI (minus sign), cPAI and nPAI matching to a PAI identified from the genome sequencing paper (circle and triangle, respectively)

When every ORF contained in 207 PAI loci (see Table 1 and supplementary Table 2S for the complete information [see Additional file 2]) were similarity-searched against the ORFs present in the 148 prokaryotic genomes, 1,490 genomic strips of PAI-associated genes were defined based on the proximity of the homologs of genes from the same PAI accession. Overlapping strips were then merged into 525 genomic regions in 83 chromosomes (Figure 1). Among these regions, 241 contained at least one gene homologous to the virulence genes on the PAI loci, which will be referred to as PAI-like regions in this study. 77 PAI-like regions (total 1,652,758 bp) partly or entirely overlapped GIs, while the remaining 164 regions (total 1,553,923 bp) did not contain any part of GIs. In this report, we call the former candidate PAIs (cPAIs). Figure 2 shows the projection of PAI-like regions in their G+C contents and length-proportion of horizontally transferred genes. 52% of all the PAI-like regions show lower G+C content compared to those of their genomes (average of -0.6%, standard deviation of 3.8), however, 75% of the cPAIs have lower G+C contents (-2.7%, 4.7, respectively). The plot indicates that clusters of PAI-homologs are often located in the backbone sequence while the detected GIs tend to be biased to have lower G+C content.

**Table 1:** A shortened list of Part of PAI loci mentioned in the text. (see supplementary Table 2S for the complete list of 207 collected PAI loci.) [see Additional file 2]

**Name**	**Function**	**Strain (abbreviation)**	**Accession number (length in kb)**^**a**^
PAI I_536_	Hemolysin, fimbriae	*Escherichia coli *536	AJ488511(77.0)^b^
PAI II_536_	Hemolysin, P fimbriae	*E. coli *536	AJ494981(102.3)^b^
PAI III_536_	S fimbriae	*E. coli *536	X16664(75.8)^b^
PAI I_CFT073_	Hemolysin, P fimbriae	*E. coli *CFT073	AF081283(10.2), AF081284, AF081285(13.7), AF081286, AF003741-2
PAI II_CFT073_	P-fimbriae	*E. coli *CFT073	AF447814(71.7)^b^
LEE	Attaching and effacing, TTSS, invasion	*E. coli *O157:H7 EDL933; E2348/69; 4797/97; 83/39; RDEC-1	AF071034(45.3)^b^, AF022236(35.6)^b^, AJ278144(37.7)^b^, AF453441(60.4)^b^, AF200363(37.9)^b^
SPI-1	TTSS, invasion into epithelial cells, apoptosis	*Salmonella typhimurium *SL1344	AF148689, U16278, U16303
SPI-2	TTSS, invasion into monocytes	*S. typhimurium *SL1344; LT2; RF333	AF020808, AJ224978(12.1), Z95891, X99944-5, AJ224892, U51927, Y09357
SPI-3	Invasion, survival in monocytes	*S. typhimurium *14028s; *S. enterica *subsp. enterica serovar Rachaburi & serovar Dublin	AF106566(17.0)^b^, Y13864, M57715, AJ000509, AY144489, AY144490(10.1)
SHI-2	Iron uptake	*S. flexneri *M90T & SA100	AF141323(23.8)^b^, AF097520(14.3)
SRL	Iron uptake	*S. flexneri *2a YSH6000	AF326777(66.7)
Yen HPI	Iron uptake	*Yersinia enterocolitica *Ye 8081 & WA314	X94452, X95298, AJ132668, AJ132945(14.0), Y12527(13.6)
Yps HPI	Iron uptake	*Y. pseudotuberculosis *PB1 & IP32637; *Y. pestis *KIM10+	AJ236887, AJ009592, AJ009988
VPI	Toxin-coregulated pilus (Tcp) adhesin, regulator	*Vibrio cholerae *395; N16961; others	AF325733(41.3)^b^, AF325734(41.3)^b^, AF034434(12.9), X64098(13.8), U39068(15.0), AF208385, AF319954, AF306795-8, AF319652-5, AF378526, AF452570-80
*cag *PAI	Type IV secretion, cytotoxing-associated gene (cag) antigen	*Helicobacter pylori*	AF282853(20.2)^b^, AF282852(21.3)^b^, U60177, AY136637-46
Hrp PAI	TTSS, effectors	*Pseudomonas syringae *DC3000 & others	AF232004(52.5)^b^, AF232005(11.0), U25812-3, AF232003, AF069650-2, L41862, U03854-5, U07346, AF051694, L11582, AY147017-28
PAGI-1		*Pseudomonas aeruginosa *X24509 & PA14	AF241171(51.3)^b^, AY273869(111.3)^b^
TTSS locus	TTSS	*P. luminescens *W14	AY144116(47.7)^b^

^a^PAI loci of < 10 kb are not listed.

^b^Fully sequenced PAI locus

### Candidate PAIs

**Figure 3:**
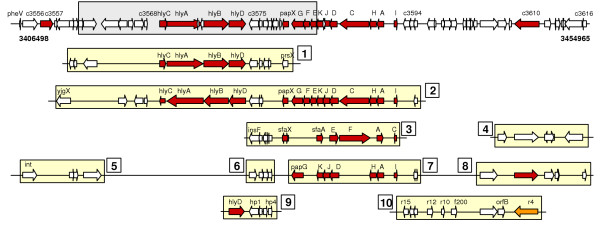
**Example of a PAI-like region and a cPAI in genome sequences. **48.5-kb of PAI I_CFT073 _from *E. coli *CFT073 was detected by merging genomic strips similar to known PAI loci (yellow strip) including partial sequence of PAI I_CFT073_. The genomic region contains homologs of the virulence genes on the known PAIs (red arrow) and genomic island (grey bar). Therefore, this PAI-like region is considered as a cPAI. Red and orange arrows in yellow strips denote virulence and putative virulence gene, respectively. Numbers on the yellow strips indicate parts of the PAI loci homologous to the genomic strips: 1. PAI I_536 _(accession number: AJ488511, host strain: *E. coli *536); 2. PAI II_536 _(AJ494981, *E. coli *536); 3. PAI III_536 _(X16664, *E. coli *536); 4. LEE (AJ278144, *E. coli *4797/97); 5 and 6. LEE (AF071034, *E. coli *O157:H7 EDL933); 7 and 8. PAI II_CFT073 _(AF447814, *E. coli *CFT073); 9. PAI I_CFT073 _(AF081284, *E. coli *CFT073); 10. PAI I_CFT073 _(AF081285, *E. coli *CFT073). Note that accessions of PAI II_CFT073 _that were included in the query set are partial sequence of the PAI. Some boxes are joined by a line for saving the space of the figure.

cPAIs, PAI-like anomalous regions, were present in 29 bacteria including 6 non-pathogens, and their sizes ranged from 3.7 kb to 137.5 kb with the average length of 21.5 kb (Table 2, supplementary Table 3S [see Additional file 3]). Most of these regions contained transposase, integrase genes or insertion sequence elements, and were associated with tRNA genes at their boundaries, which is indicative of genomic islands. In some instances, our method allowed the detection of the entire PAIs for those only partial sequences have been reported in the original papers (Figure 3). This is due to the fact that PAIs often share conserved regions, and homologous regions of other PAIs can be located in the same PAI locus. Interestingly, cPAIs were detected in six strains which are known to be non-pathogens. Genes contained code for an ABC transporter (*Bacillus halodurans*), flagellar proteins (*Bacillus subtilis*), iron transport and fimbrial proteins (*E. coli *K-12), transmembrane sensors and outer membrane efflux proteins (*Nitrosomonas europaea*), or nodulation proteins (*Bradyrhizobium japonicum*). Genes detected in *Mesorhizobium loti*, a bacterium that forms globular nodules and perform nitrogen-fixing symbiosis with leguminous plants, are involved in the nodulation process and a type III secretion system (TTSS) [13]. However, the unexpected locations of cPAIs in non-pathogens should be interpreted as some clusters of potentially horizontally transferred genes that have homology to virulence genes.

**Table 2:** PAIs in prokaryotic chromosomes (see supplementary Table 3S for the complete information) [see Additional file 3]

**Strain**	**Size (kb)**	**Δ G+C (%)**^**a**^	**HGT (%)**^**b**^	**Evidence of GI**^**c**^	**Characteristics**
*Bacillus halodurans *C-125^d^	8.1	-2.7	100.0	Transposase	ABC transporters

*Bacillus subtilis *168^d^	4.9	-2.3	13.6	-	Flagellar protein

*Bordetella bronchiseptica *RB50	15.3	-1.6	36.7	tRNA	TTSS
	8	4.3	93.2	-	Hemin transport system

*Bordetella pertussis *Tohama I	7.9	4.6	93.1	-	Heme uptake
	15.3	-1.2	39.8	tRNA	TTSS

*Bradyrhizobium japonicum *USDA 110^d^	6.4	-4.3	100.0	-	Nodulation

*Chromobacterium violaceum *ATCC 12472	11.8	-9.3	100.0	-	TTSS
	15.5	-6.5	86.4	-	TTSS

*Enterococcus faecalis *V583	137.5	-4.7	83.8	tRNA	**NN in E. faecalis**^**e**^

*Escherichia coli *CFT073	7.1	-6.3	85.8	tRNA, integrase, IS	Hypotheticals
	60.1	-1.8	33.0	tRNA, transposase, phage genes	F1C and S fimbrial protein, iron uptake
	48.5	-3.5	46.1	tRNA, integrase, transposase	**PAI I**_**CFT073**_^**e**^
	29.1	2.7	57.8	IS	ISEc8, antigen 43 precursor, fimbrial protein
	6	-9.8	87.1	-	Fimbrial protein
	43.3	-2.7	19.7	Transposase	**PAI II**_**CFT073**_^**e**^

*Escherichia coli *K12^d^	9.8	-3.8	100.0	Integrase, putative transposase	Fimbrial protein
	8.5	6.1	43.3	-	Citrate-dependent iron transport

*Escherichia coli *O157:H7 EDL933	7	-4.9	42.2	Putative transposase	Glucosyltransferase
	13.5	-4.4	100.0	-	Pilin subunit, transporter and member of exoprotein
	7	-4.9	42.1	Putative transposase, IS proteins	Glycosyl transferase, IS1 proteins
	14.9	-13.3	100.0	tRNA	TTSS
	44.7	-9.2	87.3	tRNA, integrase, phage genes	**LEE**^**e**^

*Escherichia coli *O157:H7 Sakai	7	-4.6	45.6	Transposase	Ferric enterochelin esterase
	17	-14.0	100.0	tRNA	TTSS
	44.7	-9.4	89.7	tRNA	**LEE**^**e**^

*Helicobacter pylori *26695	38	-3.0	82.6	Glutamate racemase (*glr*)	***cag*PAI**^**e**^

*Helicobacter pylori *J99	38.2	-3.1	83.7	Glutamate racemase (*murI*)	***cag*PAI**^**e**^

*Mesorhizobium loti *MAFF303099^d^	12.7	-5.5	100.0	-	TTSS, nodulation protein

*Nitrosomonas europaea *ATCC 19718^d^	16.9	2.5	34.9	Recombinase	Transmembrane sensors, outer membrane efflux

*Photorhabdus luminescens *subsp. laumondii TTO1	23.2	8.3	68.2	-	Putative fimbrial proteins
	36.3	7.7	87.4	tRNA, IS, transposase	Lipoprotein, pilus
	50.6	-1.5	17.0	-	**TTSS locus**^**e**^
	34.9	2.1	64.1	Transposase, IS	***tc *locus**^**e**^

*Salmonella enterica *Typhi Ty2	6.7	0.6	26.4	-	Fimbrial protein
	41.3	-4.7	61.2	tRNA	SPI-2^e^
	10.3	-6.5	57.7	tRNA, transposase	SPI-5^e^
	6.7	-2.4	95.5	tRNA	Fimbrial protein
	12.4	-5.2	100.0	-	SPI-1^f^
	25.5	-7.3	91.6	-	SPI-4^e^

*Salmonella enterica *Typhi CT18 (*Salmonella enterica *Typhi Typhi)	6.7	0.6	26.4	-	Fimbrial protein
	6.7	-2.5	95.5	tRNA	Fimbrial protein
	10.3	-6.6	57.7	tRNA, transposase	SPI-5^e^
	41.3	-4.7	61.2	tRNA	SPI-2^e^
	12.4	-5.2	100.0	IS, transposase	SPI-1^f^
	25.5	-7.3	91.6	-	SPI-4^e^

*Salmonella typhimurium *LT2 (*S. enterica *serovar Typhimurium LT2)	6.7	0.6	26.4	-	Fimbrial protein
	8.3	-3.5	77.4	-	Fimbrial protein
	9.5	-6.6	52.2	tRNA	**SPI-5**^**e**^
	41.6	-4.7	60.9	tRNA	**SPI-2**^**e**^
	15.1	0.6	100.0	Putative transposase	Flagellar synthesis, siderophore receptor protein
	12.4	-5.3	100.0	-	**SPI-1**^**f**^
	18	-4.5	56.8	tRNA	**SPI-3**^**e**^
	25.5	-7.5	97.8	-	**SPI-4**^**e**^

*Shigella flexneri *2a 2457T	50.1	-1.9	17.2	tRNA	**SHI-1**^**e**^
	25	-2.3	31.5	tRNA	**SHI-2**^**e**^
	22.6	-4.1	48.0	tRNA, recombinase	Fimbrial protein

*Shigella flexneri *2a 301	13.7	1.8	83.6	Putative transposase	Enterochelin esterase, oxidoreductase (Fe-S subunit)
	7.5	-3.1	54.8	tRNA	Oxidoreductases (Fe-S subunit)
	53.5	-2.1	8.1	tRNA, integrase, transposase	**SHI-1**^**e**^
	28.1	-2.5	48.2	tRNA, integrase, transposase	**SHI-2**^**e**^
	28.9	-3.4	50.1	tRNA, transposase, integrase	Fimbrial protein

*Staphylococcus aureus *Mu50	5.3	-7.0	100.0	tRNA	SaPIm3^f^

*Staphylococcus aureus *MW2	6.3	0.4	43.8	-	ν Saβ^g^

*Staphylococcus aureus *N315	5.3	-6.9	100.0	tRNA	SaPIn3^f^

*Vibrio cholerae *N16961	4.3	-11.5	87.9	Transposase	**VPI**^**e**^
	8.8	-3.2	100.0	-	**CTX locus**^g^

*Vibrio parahaemolyticus *RIMD 2210633 chromosome I	16.7	2.9	39.6	-	TTSS
	11.3	0.0	11.1	-	TTSS, iron transport

*Vibrio parahaemolyticus *RIMD 2210633 chromosome II	9	0.5	26.1	-	Flagellar biosynthesis
	3.7	4.9	79.2	-	Iron transport

*Xanthomonas campestris *pv. campestris ATCC 33913	23.1	-1.8	10.0	Transposase	**Hrp PAI**^**e**^

*Yersinia pestis *CO92	34.7	9.1	73.1	tRNA, integrase	**HPI**^**e**^
	8	-1.7	48.7	Transposase	Iron transport system
	6.1	-0.9	100.0	Transposase	Fimbrial protein, secreted protein

*Yersinia pestis *KIM	34.7	9.1	76.8	tRNA, integrase	**HPI**^**e**^
	14.6	-0.6	5.6	-	Iron/siderophore ABC transporters, antigen chaperone

^a^Deviation of the G+C content of the cPAI as compared to that of the whole genome

^b^Length percentage of horizontally transferred genes in the cPAI

^c^Genes involved in the transfer mechanism (integrase, transposase, IS element, or tRNA gene at the boundaries)

^d^Non-pathogenic bacterium

^e^cPAI that entirely matches to a PAI identified from the genome sequencing paper

^f^cPAI that matches to one end of a PAI identified from the genome sequencing paper. The other end of the PAI is present in a PAI-like region not overlapping GIs.

^g^cPAI that partly matches to a PAI identified from the genome sequencing paper

Bold characters denote that a sequenced strain containing the cPAI is the same as or closely related to the host strain of the queried PAI loci.

Among the 77 cPAIs, 34 matched to PAIs which have been described in genome sequencing papers (Table 2, Figure 2). 27 cPAIs entirely matched to known PAIs – a PAI (in *Enterococcus faecalis*), PAI I, II_CFT073 _(*E. coli *CFT073), LEE (*E. coli *O157 EDL933 and Sakai), *cag *PAI (*Helicobacter pylori *26695 and J99), the TTSS and *tc *loci (*Photorhabdus luminescens*), SPI-2,4,5 (*Salmonella enterica *serovar Typhi Ty2 and CT18, and serovar Typhimurium LT2), SPI-3 (*S. typhimurium *LT2), SHI-1, 2 (*Shigella flexneri *2a 2457T and 301), VPI (*Vibrio cholerae*), Hrp PAI (*Xanthomonas campestris*), and HPI (*Yersinia pestis *CO92 and KIM). One end of PAIs – SPI-1 (in three *S. enterica *strains), SaPIm3 (*S. aureus *Mu50), and SaPIn3 (*S. aureus *N315) – were found in 5 cPAIs, and the other end of the PAIs were found in seemingly backbone sequences. νSaβ in *S. aureus *MW2 and CTX locus in *V. cholerae *N16961 were partly matched. Nine cPAIs span the TTSS loci which were not annotated as PAIs in the genome sequencing data.

**Figure 4:**
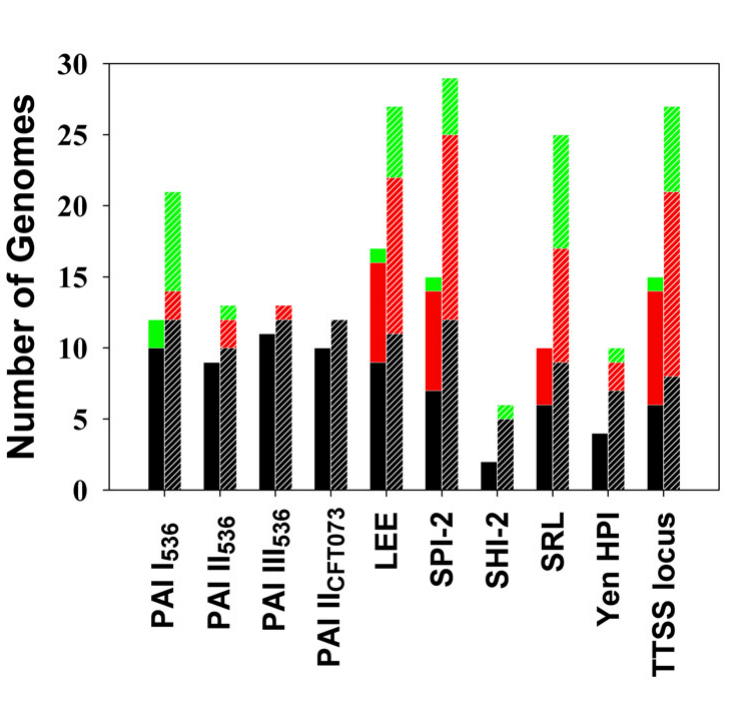
**Distribution of genomic regions homologous to the PAIs from enteropathogenic bacteria. **According to each PAI, left bar denotes the number of genomes containing at least one cPAI. Right hatched bar delineates the number of genomes containing at least one PAI-like region. Different colors represent the number of genomes of different taxon – Enterobacteriales (black), Proteobacteria except Enterobacteriales (red), and phylums except Proteobacteria (green). The demonstrated PAIs are PAI I,II,III_536 _in uropathogenic *E. coli *536, PAI II_CFT073 _in uropathogenic *E. coli *CFT073, LEE in enterohemorrhagic *E. coli *O157, SPI-2 in *S. typhimurium*, SHI-2 and SRL in *S. flexneri*, HPI in *Y. enterocolitica*, and TTSS locus in *Photorhabdus lumniescens*.

Regions homologous to a certain PAI were frequently found in genomes of various taxa. Especially, parts of PAIs originally identified from enteropathogenic bacteria were detected not only in enterobacteria but also in phyla other than the Gammaproteobacteria in our study (Figure 4). The number of genomes containing PAI-like regions was drastically reduced when we considered genomic regions that overlap GIs. Elements of PAI I~ III_536 _in the uropathogenic *E. coli *strain 536 showed high similarities to other members of the *Enterobacteriaceae*. This is consistent with the previous report that PAI-specific sequences of *E. coli *strain 536 were frequently found in pathogenic and commensal *E. coli *isolates by using "*E. coli *pathoarray" [14]. Parts of the LEE PAI in enterohemorrhagic *E. coli *O157:H7, enteropathogenic *E. coli *E2348/69, rabbit-specific enteropathogenic *E. coli *83/89, and rabbit diarrheagenic *E. coli *RDEC-1 similarly matched to genomic regions of different taxa.

In most cases, distribution of the regions homologous to the PAIs from other enterobacteria such as VPI of *Vibrio cholerae*, *cag *PAI of *Helicobacter pylori*, SaPI1 of *Staphylococcus aureus *strains were restricted to their host strains. However, widespread distribution in different species was evident for PAGI-1 of *Pseudomonas aeruginosa *and the Hrp PAI of *P. syringae*, *Xanthomonas *spp., *Burkholderia pseudomallei*, and *Ralstonia solanacearum*. Variations of cPAIs were observed for EDL933 and Sakai, which belong to the same *E. coli *O157 group (Table 2). This discrepancy results from the different distribution of prophages in the two genomes. Also, different ORF prediction by different research groups affected the determination of GIs.

### PAI-like regions that did not meet the criteria

164 PAI-like regions in 57 prokaryotes including 16 non-pathogenic bacteria and one archaeon did not overlap GIs (supplementary Table 4S) [see Additional file 4]. Their sizes ranged from 1.9 to 50.6 kb and were averaged 9.5 kb. Most of them encoded flagellar/fimbrial biosynthesis or iron uptake systems. Among these regions, 14 were PAIs published in the genome sequencing papers. Six PAIs – Hrp PAI (in *Pseudomonas syringae *pv. tomato DC3000), SPI-3 (*S. enterica *serovar Typhi strains Ty2 and CT18), SaPIm1 (in *S. aureus *Mu50), SaPIn1 (*S. aureus *N315) and νSa3 (*S. aureus *MW2) – entirely matched, and 5 counterparts of the PAIs that partly match to the cPAIs that overlap GIs were found in these regions. Parts of LIPI-1 in *Listeria innocua *and two regions of internalins in *L. monocytogenes *EGD were found. In fact, the Hrp PAI and LIPI-1 have DNA compositions similar to the core genomes, and are suggested to have been acquired a long time ago [15,16].

## Discussion

By analyzing structures of many microbial genomes, it became obvious that HGT is an important mechanism for bacterial evolution, let alone genome complexity and plasticity [1]. GIs, which are large genomic segments and most likely transferred by HGT, contribute to the survival of the hosting bacterial strain in a particular environment and sometimes to virulence. These two kinds of GIs, of which the former can be referred as 'fitness islands', are often hardly distinguishable from each other because the role of a GI may vary in different ecological niches and the physiology of the bacterium. Up to now, attempts to identify PAIs [5,6,17] have been made by detecting genomic regions which only differ from the rest of the genome in their base composition and codon usage. In this study, we identified "candidate PAIs (cPAIs)" that reflect potential PAIs with anomalous composition, probably due to their recent acquisition. Among the 148 sequenced strains searched in this study, 17 were the strains closely related to the hosts carrying queried PAI loci. From the reports of their genome sequencing projects, 27 PAIs have been described. Among them, 23 PAIs were found in the list of cPAIs and the accuracy of our method can be considered as 85% (Table 2, supplementary Table 4S [see Additional file 4]).

The presence of virulence factors could be a useful criterion for discerning PAIs from other genomic islands. Clusters consisting of only hypothetical genes and/or elements involved in the transfer mechanism (e.g. IS elements, tRNA genes, integrase, and prophage) were filtered out, leaving only 46% of the genomic regions containing virulence factors. Widespread distribution of conserved elements of many PAIs in different species and in even non-pathogens is due to their complex mosaic structures consisting of elements of different origins. PAI I~ III_536 _in *E. coli *536 have mosaic-like structures consisting of many DNA fragments that show high similarities to the chromosomal regions of other pathogenic *E. coli *strains and *Shigella flexneri*[18]. SPI-2 is a fusion of at least two genetic elements – a 25-kb region encoding the TTSS with a low G+C content and a 15-kb region encoding metabolic functions with a G+C content similar to the rest of the genome [19], and the Hrp PAI of *Pseudomonas syringae *has a tripartite structure [15].

Some virulence factors in PAIs are homologous to seemingly backbone genes. As shown in Figure 4, PAIs having extensive mosaic structures showed highly frequent occurrence in various species, and clusters of seemingly backbone genes could be removed from the list of the cPAIs by checking the presence of a GI in a PAI-like region. Many Gram-negative bacterial pathogens cause diseases by secreting and injecting virulence proteins (effectors) into the host cell via a specialized protein secretion mechanism (TTSS) [20]. They are evolutionarily related to flagellar systems and often hard to distinguish when based only on homology searches [21]. However, TTSSs are frequently transferred laterally between Gram-negative bacteria while flagellar systems are mainly inherited by vertical descent. This fact explains why many regions encoding flagellar biosynthesis genes have hits to PAI-like regions not showing anomalies in DNA composition (supplementary Table 4S) [see Additional file 4], while PAI-like regions overlapping GIs contain lots of TTSSs (Table 2). Iron uptake systems are important for bacterial survival as well as virulence [2]. Many PAIs such as HPI of *Yersinia *species, SHI-2 of *S. flexneri*, and SRL of *S. flexneri *2a YSH6000 carry genes encoding various siderophore systems that produce and secrete low-molecular-weight siderophores with extremely high affinities for ferric iron. Clusters of homologs of ferric dicitrate transport system (*fecABCDEIR*, Fec) of SRL [22] were widely distributed in the backbone genomic regions of various species, which implies that Fec might be the most ancient siderophore system (Figure 4, Table 2, supplementary Table 4S [see Additional file 4]). Interestingly, a 7.1-kb *fecCDE*-homologous region can be found even in *Halobacterium *sp. NRC-1, the only archaeon possessing the PAI-like region in this study. This region is inserted by a 6-phosphogluconate dehydrogenase gene, 3 hypothetical proteins and tRNA-Arg gene.

One of the difficulties when dubbing potential PAIs in the sequenced genomes is to determine the boundaries. A PAI may have a number of genes which have undergone many evolutionary stages and thus compositionally indistinguishable from the rest of the genome [2,23]. This might be due to some parts highly adjusted to the base composition of the recipient's genome or to the backbone genomic segments added later in evolution [10]. We found that the length proportion of transferred regions contained in the known chromosomal PAIs – 28.7 kb of LEE in *E. coli *O157 Sakai, 36.2 kb of Cag PAI in *H. pylori *26695, 61.2 kb of VPI-2 in *V. cholerae*, and 137.5 kb of PAI in *Enterococcus faecalis *– vary from 0.19 to 0.65. Thus, compositional approaches cannot predict the boundaries of the detected PAI because they only detect atypical genomic region. To solve this problem, we detected genomic segments homologous to each known PAI, which were then clumped into a large genomic region. This procedure is somewhat like the process of fragment assembly in which a contiguous region (contig) is made from overlapping fragments in shotgun sequencing [24]. Like the conserved sequences of TTSS structural genes [20], PAIs often share conserved regions. In addition, PAIs frequently carry relics of HGT event such as mobile sequence elements and association with tRNA genes at their boundaries [3]. Islander [25], a database of potential integrative islands in prokaryotic genomes, detects GIs by identifying tRNAs or tmRNA genes, and candidate integrase genes. Although many GIs reported from the database were in accordance with our results, large portion was not annotated as cPAIs mainly due to the absence of homologs of virulence genes in known PAIs and PAIs that are not located at the tRNA loci. As illustrated in Figure 3, frequent distribution of conserved regions between PAIs allows our method to find the entire region of a PAI in a sequenced genome even though its similar sequence is partially known.

A typical genome sequencing team uses genes in the gene cluster or the genome sequence of interest as a query to search for any similar genes in the databases. Then, homologs of pathogenicity/virulence genes are inferred by checking whether descriptions of the retrieved genes have any indications that suggest virulence/pathogenicity or they are from pathogens. Because this approach depends on the examiner's knowledge on known PAIs or pathogenicity/virulence genes and entry descriptions of the retrieved genes often are not informative to infer the function, it is never sure whether the searches thoroughly picked up all the genes associated with PAIs or pathogenicity/virulence. To avoid this uncertainty on the robustness of the open-ended search, we first collected all the reported PAI loci and used them as a query to search for homologs in the complete prokaryotic genomes. Our method guarantees that all the potential PAIs related to the known PAIs were searched without the intervention of human interpretation.

In completely sequenced genomes, we detected cPAIs that are homologous to the published PAIs and show anomaly in DNA composition. The methodology we developed in this study has a limitation in that the detected cPAIs are limited by the query data set of the known PAIs. This caveat, however, can be advantageous when the researchers only concern a specific set of PAIs. Furthermore, this approach can be easily extended to identify various genomic islands (e.g. fitness, metabolism, and resistance islands). Among the cPAIs detected in this study, omission of several well-known PAIs such as Hrp PAI of *P. syringae *and LIPI-1 of *L. innocua *is due to their DNA compositions similar to the core genomes which may caused by horizontal transfer from closely related strains or very ancient HGT event. Thus, patterns of best matches of each gene to different species, lineage-specific genes or transferred genes from phylogenetically distant species would be helpful in improving the possibility of finding GIs and PAIs. Also, accumulation of PAI sequence data in bacterial families other than the *Enterobacteriaceae *will lead to detection of more putative PAIs across various taxa. Finally, it should be noted that the identity of cPAIs as bona fide PAIs need to be confirmed by further experimental verification. We are currently improving the detection scheme and are developing a database for cPAIs in sequenced genomes.

## Conclusion

We present the first computational framework combining feature-based analyses and similarity-based analyses. As shown in Figure 3, the similarity-based analysis that is reminiscent of the sequence-assembly procedure was proven to be an efficient method for demarcating the potential PAIs in our study. Also, the function(s) and origin(s) of a cPAI can be inferred by investigating the PAI queries comprising it. With the availability of rapidly increasing complete genome sequences [26] as well as PAI data, the proposed method will be useful in identifying potential PAIs in microbial genomes.

## Methods

### Collection of complete genomes and PAI Data

The sequence files of 148 prokaryotic complete genomes consisting of 157 chromosomes, including 17 archaeal ones as of January 2004 were downloaded from the NCBI FTP server (, supplementary Table 1S) [see Additional file 1]. We searched the GenBank database and literature [3,23] for any descriptions of the "pathogenicity island". Forty five kinds of PAIs and 207 GenBank accessions containing either part or all of the reported PAI loci in 120 pathogenic bacteria, are summarized in Table 1. (see supplementary Table 2S for the complete information) [see Additional file 2]. The definition of virulence genes is difficult as their function may depend on growth conditions and host niches. Thus, we attributed this to the biologists who identified PAI loci, and virulence genes of PAI loci were identified by literature survey. Many PAIs, 29 out of 45 kinds of PAIs, came from *Enterobacteriaceae*. Thirty four PAI loci are completely sequenced ones ranging from 6.8 kb to 153.6 kb (average: 41.3 kb), and the remains are part of PAI. It should be noted that the collected sets do not contain PAIs which were reported from genome sequencing papers.

### Detection of GIs in genome sequences

To detect GIs in a chromosome, we first identified horizontally transferred genes (H) based on the algorithm developed by Garcia-Vallve et al. [4]. To alleviate false positives caused by applying single criterion for identifying HGT regions, we considered a gene as H only if both G+C content and codon usage are aberrant. For each genome, we have computed total G+C content ([G+C]_T_) and G+C contents at the first and third codon positions ([G+C]_1 _and [G+C]_3_) of every ORF. The compositional bias at the first and third positions were reported to be positively correlated to expressivity and genomic G+C content, respectively [10,27]. Extraneous origin of the gene in terms of G+C content was considered if its [G+C]_T _deviates over 1.5 σ or if deviations of [G+C]_1 _and [G+C]_3 _are of the same sign and at least one of them is over 1.5 σ. Mahalanobis distance (d^M^) was used to evaluate deviation of the codon usage of a gene and mean of the genome [4]. d^M ^is a statistic in unit of standard deviation from the mean of 61 codon frequencies and can be calculated as follows:

*d*^*M*^*(X, X*_*mean*_*) *= *(X *- *X*_*mean*_*)*^*T *^*S*^-1^*(X *- *X*_*mean*_*)*

Where X and X_mean _correspond to vectors having relative frequencies of the 61 codons for a gene and the mean values for a genome, respectively. S^-1 ^is the inverse of variance-covariance matrix (S) of all the 61 codon frequencies. The higher this value is the more deviation in codon usage [4]. If Xs are normally distributed, d^M^s can be converted to p-values using the χ^2 ^distribution function. We considered a gene as extraneous in codon usage if its p-value was less than 0.05. It should be noted that genes longer than 300 bp were used for calculating the mean and standard deviation (σ) of G+C contents and d^M^s. This is from the observation that genes having shorter than 300 bp have much higher chance of anomalies in G+C content and codon usage.

We ran a genome scan of a 10-gene window and identified regions containing four or more H. This threshold frequency of 0.4 was inferred from the observation that the frequencies of H in known PAIs such as LEE of *E. coli *O157 Sakai, *cag *PAI of *Helicobacter pylori *26695, VPI-2 of *Vibrio cholerae*, and a PAI of *Enterococcus faecalis*, were averaged 0.35. Neighbouring regions were merged into larger regions which were referred to as GIs in this study. Some genomic regions had highly biased G+C content compared to the whole G+C content of the chromosome, while their codon usage were not biased. For example, 46.4 kb genomic region ranging from 2,647,129 bp in *Yersinia pestis *KIM, which contains yersiniabactin genomic island [28] has considerably higher G+C content (55.7% versus 47.6% average for the whole genome), but showed a similar codon usage for the genes contained in this region. Thus, among genomic regions made from genes anomalous in G+C content, the region was added to GIs if its G+C(T) deviates more than 1.5 σ.

### Identification of candidate PAIs

The detection scheme for the regions of cPAIs is outlined in Figure 1. Each ORF from PAI locus was used as the query in BLASTP searches [29] against the set of ORFs from each of the 148 completely sequenced genomes using PAM250 as scoring matrix for retrieving homologous genes in evolutionary distant strains. Likewise, homologs of ORFs, RNA genes and repeat regions of PAI locus on the nucleotide level were searched using BLAT, a modified BLAST alignment program which can stitch matched regions into a larger one [30]. If the identity of the resulting hit is over 80% for DNA sequence or 25% for protein sequence and the aligned region is both over 70% of lengths of query and the hit, the pair of sequences was considered as a homolog. Genomic strips corresponding to each PAI locus were then obtained by identifying the regions containing four or more homologs of the genes from the same PAI accession and by merging the neighboring regions. Overlapping or adjacent genomic strips corresponding to the same or different kind of PAI loci were fused into a large region. Among these regions, PAI-like regions were identified by checking the presence of at least one gene homologous to a virulence gene on the PAI loci. We considered a candidate PAI (cPAI) only if the PAI-like region partly or entirely spans the GI.

## Authors' contributions

SHY designed the study, developed the software for implementing the devised algorithm, and wrote the manuscript. CH and HK contributed to the writing the software, and YHK collected and reviewed the data, and TKO assessed the biological significance of the results. JFK supervised the project and contributed to the development of methodology and writing the manuscript. All authors read and approved the final manuscript.

## Supplementary Material

Additional File 1 The complete list of organisms whose genomes were searched for candidate PAIs in this study Click here for file

Additional File 2 Complete list of the PAI loci used as the query for BLASTP searches Click here for file

Additional File 3 Detailed information of candidate PAIs in prokaryotic chromosomes Click here for file

Additional File 4 Detailed information of PAI-like regions not overlapping GIs in prokaryotic chromosomes Click here for file
