# Supplementary material for: A computational approach for identifying pathogenicity islands in prokaryotic genomes
Source: BMC Bioinformatics. 2005 Jul 21;6:184. doi: 10.1186/1471-2105-6-184 (PMC1188055; doi:10.1186/1471-2105-6-184)
Supplement: Additional File 1 — The complete list of organisms whose genomes were searched for candidate PAIs in this study [file 1471-2105-6-184-S1.doc]

Table 1S. Organisms whose genomes were searched for candidate PAIs in this study

| **Organism** | **Classification^a^** | **Organism** | **Classification^a^** |
| --- | --- | --- | --- |
| *Aeropyrum pernix* K1 | Archaea | *Mycoplasma pneumoniae* M129 | Firmicutes |
| *Agrobacterium tumefaciens* C58 (Cereon) | Alphaproteobacteria | *Mycoplasma pulmonis* | Firmicutes |
| *Agrobacterium tumefaciens* C58 (U. Washington) | Alphaproteobacteria | *Nanoarchaeum equitans* Kin4-M | Archaea |
| *Aquifex aeolicus* VF5 | Aquificae (N) | *Neisseria meningitidis* MC58 | Betaproteobacteria |
| *Archaeoglobus fulgidus* DSM 4304 | Archaea | *Neisseria meningitidis* Z2491 | Betaproteobacteria |
| *Bacillus anthracis* A2012 | Firmicutes | *Nitrosomonas europaea* ATCC 19718 | Betaproteobacteria (N) |
| *Bacillus anthracis* Ames | Firmicutes | *Nostoc* sp. PCC 7120 | Cyanobacteria (N) |
| *Bacillus cereus* ATCC 14579 | Firmicutes | *Oceanobacillus iheyensis* HTE831 | Firmicutes (N) |
| *Bacillus halodurans* C-125 | Firmicutes (N) | Onion yellows *phytoplasma* onion yellows | Firmicutes |
| *Bacillus subtilis* 168 | Firmicutes (N) | *Pasteurella multocida* PM70 | Gammaproteobacteria |
| *Bacteroides thetaiotaomicron* VPI-5482 | Bacteroidetes (N) | *Photorhabdus luminescens* subsp. laumondii TTO1 | Enterobacteriales |
| *Bifidobacterium longum* NCC2705 | Actinobacteria (N) | *Pirellula* sp. 1 | Planctomycetes (N) |
| *Bordetella bronchiseptica* RB50 | Betaproteobacteria | *Porphyromonas gingivalis* W83 | Bacteroidetes |
| *Bordetella parapertussis* 12822 | Betaproteobacteria | *Prochlorococcus marinus* MIT9313 | Cyanobacteria (N) |
| *Bordetella pertussis* Tohama I | Betaproteobacteria | *Prochlorococcus marinus* subsp. marinus str. CCMP1375 | Cyanobacteria (N) |
| *Borrelia burgdorferi* B31 | Spirochaetes | *Prochlorococcus marinus* subsp. pastoris str. CCMP1986 | Cyanobacteria (N) |
| *Bradyrhizobium japonicum* USDA 110 | Alphaproteobacteria (N) | *Pseudomonas aeruginosa* PA01 | Gammaproteobacteria |
| *Brucella melitensis* 16M | Alphaproteobacteria | *Pseudomonas putida* KT2440 | Gammaproteobacteria (N) |
| *Brucella suis* 1330 | Alphaproteobacteria | *Pseudomonas syringae* pv. tomato DC3000 | Gammaproteobacteria |
| *Buchnera aphidicola* APS | Enterobacteriales (N) | *Pyrobaculum aerophilum* IM2 | Archaea |
| *Buchnera aphidicola* Bp | Enterobacteriales (N) | *Pyrococcus abyssi* | Archaea |
| *Buchnera aphidicola* Sg | Enterobacteriales (N) | *Pyrococcus furiosus* DSM 3638 | Archaea |
| *Campylobacter jejuni* NCTC 11168 | Epsilonproteobacteria | *Pyrococcus horikoshii* OT3 | Archaea |
| *Candidatus Blochmannia* floridanus | Enterobacteriales (N) | *Ralstonia solanacearum* GMI1000 | Betaproteobacteria |
| *Caulobacter crescentus* CB15 | Alphaproteobacteria (N) | *Rhodopseudomonas palustris* CGA009 | Alphaproteobacteria (N) |
| *Chlamydia muridarum (Chlamydia trachomatis* MoPn*)* | Chlamydiae | *Rickettsia conorii* Malish 7 | Alphaproteobacteria |
| *Chlamydia trachomatis* D/UW-3/CX | Chlamydiae | *Rickettsia prowazekii* Madrid E | Alphaproteobacteria |
| *Chlamydophila caviae* GPIC | Chlamydiae | *Salmonella enterica* Typhi CT18 | Enterobacteriales |
| *Chlamydophila pneumoniae* AR39 | Chlamydiae | *Salmonella enterica* Typhi Ty2 | Enterobacteriales |
| *Chlamydophila pneumoniae* CWL029 | Chlamydiae | *Salmonella typhimurium* LT2 | Enterobacteriales |
| *Chlamydophila pneumoniae* J138 | Chlamydiae | *Shewanella oneidensis* MR-1 | Gammaproteobacteria (N) |
| *Chlamydophila pneumoniae* TW-183 | Chlamydiae | *Shigella flexneri* 2a 2457T | Enterobacteriales |
| *Chlorobium tepidum* TLS | Chlorobi | *Shigella flexneri* 2a 301 | Enterobacteriales |
| *Chromobacterium violaceum* ATCC 12472 | Betaproteobacteria | *Sinorhizobium meliloti* | Alphaproteobacteria (N) |
| *Clostridium acetobutylicum* ATCC824 | Firmicutes (N) | *Staphylococcus aureus* Mu50 | Firmicutes |
| *Clostridium perfringens* 13 | Firmicutes | *Staphylococcus aureus* MW2 | Firmicutes |
| *Clostridium tetani* E88 | Firmicutes | *Staphylococcus aureus* N315 | Firmicutes |
| *Corynebacterium diphtheriae* NCTC13129 | Actinobacteria | *Staphylococcus epidermidis* ATCC 12228 | Firmicutes |
| *Corynebacterium efficiens* YS-314 | Actinobacteria (N) | *Streptococcus agalactiae* 2603V/R | Firmicutes |
| *Corynebacterium glutamicum* ATCC 13032 | Actinobacteria (N) | *Streptococcus agalactiae* NEM316 | Firmicutes |
| *Coxiella burnetii* RSA 493 | Gammaproteobacteria | *Streptococcus mutans* UA159 | Firmicutes |
| *Deinococcus radiodurans* R1 | Deinococcus-Thermus (N) | *Streptococcus pneumonia* R6 | Firmicutes |
| *Enterococcus faecalis* V583 | Firmicutes | *Streptococcus pneumonia* TIGR4 | Firmicutes |
| *Escherichia coli* CFT073 | Enterobacteriales | *Streptococcus pyogenes* MGAS315 | Firmicutes |
| *Escherichia coli* K12 | Enterobacteriales (N) | *Streptococcus pyogenes* MGAS8232 | Firmicutes |
| *Escherichia coli* O157:H7 EDL933 | Enterobacteriales | *Streptococcus pyogenes* SF370 | Firmicutes |
| *Escherichia coli* O157:H7 Sakai | Enterobacteriales | *Streptococcus pyogenes* SSI-1 | Firmicutes |
| *Fusobacterium nucleatum* ATCC 25586 | Fusobacteria | Streptomyces avermitilis MA-4680 | Actinobacteria(N) |
| *Geobacter sulfurreducens* PCA | Deltaproteobacteria (N) | *Streptomyces coelicolor* A3(2) | Actinobacteria (N) |
| *Gloeobacter violaceus* PCC 7421 | Cyanobacteria (N) | *Sulfolobus solfataricus* P2 | Archaea |
| *Haemophilus ducreyi* 35000HP | Gammaproteobacteria | *Sulfolobus tokodaii* strain7 | Archaea |
| *Haemophilus influenzae* Rd KW20 | Gammaproteobacteria | *Synechococcus* sp. WH8102 | Cyanobacteria (N) |
| *Halobacterium* sp. NRC-1 | Archaea | *Synechocystis* sp. PCC6803 | Cyanobacteria (N) |
| *Helicobacter hepaticus* ATCC 51449 | Epsilonproteobacteria | *Thermoanaerobacter tengcongensis* MB4T | Firmicutes (N) |
| *Helicobacter pylori* 26695 | Epsilonproteobacteria | *Thermoplasma acidophilum* | Archaea |
| *Helicobacter pylori* J99 | Epsilonproteobacteria | Thermoplasma volcanium GSS1 | Archaea |
| *Lactobacillus plantarum* WCFS1 | Firmicutes (N) | *Thermosynechococcus elongatus* BP-1 | Cyanobacteria (N) |
| *Lactococcus lactis* IL1403 | Firmicutes (N) | *Thermotoga maritima* MSB8 | Thermotogae (N) |
| *Leptospira interrogans* 56601 | Spirochaetes | *Treponema pallidum* subsp*. pallidum* Nichols | Spirochaetes |
| *Listeria innocua* | Firmicutes (N) | *Tropheryma whipplei* TW08/27 | Actinobacteria |
| *Listeria monocytogenes* EGD | Firmicutes | *Tropheryma whipplei* Twist | Actinobacteria |
| *Mesorhizobium loti* MAFF303099 | Alphaproteobacteria (N) | *Ureaplasma urealyticum* serovar 3 | Firmicutes |
| *Methanococcus jannaschii* | Archaea | *Vibrio cholerae* N16961 | Gammaproteobacteria |
| *Methanopyrus kandleri* AV19 | Archaea | *Vibrio parahaemolyticus* RIMD 2210633 | Gammaproteobacteria |
| *Methanosarcina acetivorans* C2A | Archaea | *Vibrio vulnificus* CMCP6 | Gammaproteobacteria |
| *Methanosarcina mazei* Goe1 | Archaea | *Vibrio vulnificus* YJ016 | Gammaproteobacteria |
| *Methanothermobacter thermautotrophicus* Delta H | Archaea | *Wigglesworthia glossinidia* brevipalpis | Enterobacteriales |
| *Mycobacterium bovis* AF2122/97 | Actinobacteria | *Wolinella succinogenes* DSMZ 1740 | Epsilonproteobacteria (N) |
| *Mycobacterium leprae* TN | Actinobacteria | *Xanthomonas axonopodis* pv. citri 306 | Gammaproteobacteria |
| *Mycobacterium tuberculosis* CDC1551 | Actinobacteria | *Xanthomonas campestris* pv. campestris ATCC 33913 | Gammaproteobacteria |
| *Mycobacterium tuberculosis* H37Rv | Actinobacteria | *Xylella fastidiosa* 9a5c | Gammaproteobacteria |
| *Mycoplasma gallisepticum* R | Firmicutes | *Xylella fastidiosa* Temecula1 | Gammaproteobacteria |
| *Mycoplasma genitalium* | Firmicutes | *Yersinia pestis* CO92 | Enterobacteriales |
| *Mycoplasma penetrans* HF-2 | Firmicutes | *Yersinia pestis* KIM | Enterobacteriales |

^a^N denotes pathogenic bacterium.

Alphaproteobacteria, Betaproteobacteria, Deltaproteobacteria, Epsilonproteobacteria, and Gammaproteobacteria are subdivision of Proteobacteria.

Enterobacteriales denotes for Enterobacteriales of Gammaproteobacteria.

Gammaproteobacteria notes for Gammaproteobacteria other than Enterobacteriales.
