# Supplementary material for: A computational approach for identifying pathogenicity islands in prokaryotic genomes
Source: BMC Bioinformatics. 2005 Jul 21;6:184. doi: 10.1186/1471-2105-6-184 (PMC1188055; doi:10.1186/1471-2105-6-184)
Supplement: Additional File 2 — Complete list of the PAI loci used as the query for BLASTP searches [file 1471-2105-6-184-S2.doc]

Table 2S. Complete list of the PAI loci used as the query for BLASTP searches

| **Name** | **Function** | **Strain (abbreviation^a^)** | **Accession number (length in kb)^b^** |
| --- | --- | --- | --- |
| PAI I_536_ | Hemolysin, fimbriae | *Escherichia coli* 536 (UPEC) | AJ488511(77.0)^c^ |
| PAI II_536_ | Hemolysin, P fimbriae | *E. coli* 536 (UPEC) | AJ494981(102.3)^c^ |
| PAI III_536_ | S fimbriae | *E. coli* 536 (UPEC) | X16664(75.8)^c^ |
| PAI IV_536_ | Iron uptake | *E. coli* 536 (UPEC) | AF135406, AF136296 |
| PAI I_CFT073_ | Hemolysin, P fimbriae | *E. coli* CFT073 (UPEC) | AF081283(10.2), AF081284, AF081285(13.7), AF081286, AF003741-2 |
| PAI II_CFT073_ | P-fimbriae | *E. coli* CFT073 (UPEC) | AF447814(71.7)^c^ |
| NN^d^ | Uropathogenic-specific protein (USP) | UPEC strains | AB056434(13.2), AB056435-40 |
| PAI I | Afimbrial adhesin | *E. coli* AL862 | AF072900, AF286670, AF286671 |
| LEE | Attaching and effacing, TTSS, invasion | *E. coli* O157:H7 EDL933 (EHEC); E2348/69 (EPEC); 4797/97 (STEC); 83/39 (REPEC); RDEC-1 | AF071034(45.3)^c^, AF022236(35.6)^c^, AJ278144(37.7)^c^, AF453441(60.4)^c^, AF200363(37.9)^c^ |
| LEE | Attaching and effacing, TTSS, invasion | *E. coli* 84/110-1 (REPEC); RW1374 (STEC); 172 (STEC) | AF453442(10.7), AF461393-4, AJ303141(10.8), AJ303142(20.1), AF041809-10 |
| LEE | Attaching and effacing, TTSS, invasion | *Citrobacter rodentium* DBS100 | AF311901(42.0)^c^ |
| *espC* PAI | Enterotoxin | *E. coli* E2348/69 (EPEC) | AF297061(5.2)^c^ |
| NN^d^ | Labile enterotoxin output (leo) | *E. coli* H10407 | AF170971 |
| LIPI-1 | Internalin | *Listeria ivanovii* ATCC 19119 (Liv); *Listeria monocytogenes* (Lmo) P14; F2365; LO28 | X72685, X81135, AJ409322-3, AJ002742, U25443-4, M82881 |
| LIPI-2 | Internalin | *L. ivanovii* ATCC 19119 (Liv) | AJ004808(10.3), Y09477, Y09988, AJ251980-1, AJ271621, Y07639 |
| Internalin | Internalin | *L. monocytogenes* EGD (Lmo) | AJ012346, U77367-8, AJ012385, AJ007319, Y07640 |
| SPI-1 | TTSS, invasion into epithelial cells, apoptosis | *Salmonella typhimurium* SL1344 (Sty) | AF148689, U16278, U16303 |
| SPI-2 | TTSS, invasion into monocytes | *S. typhimurium* SL1344; LT2; RF333 (Sty) | AF020808, AJ224978(12.1), Z95891, X99944-5, AJ224892, U51927, Y09357 |
| SPI-3 | Invasion, survival in monocytes | *S. typhimurium* 14028s (Sty); *S. enterica* subsp. enterica serovar Rachaburi & serovar Dublin (Sen) | AF106566(17.0)^c^, Y13864, M57715, AJ000509, AY144489, AY144490(10.1) |
| SPI-4 | Invasion, survival in monocytes | *S. typhimurium* LT2 & ST4/74 (Sty) | AF060869(27.3), AJ576316(24.7) |
| SPI-5 | Effectors for SPI-1 & SPI-2 | *S. enterica* various (Sen); *Salmonella bongori* SARC11 (Sbo) | AF060858(9.7)^c^, AY144491-2, AF323077-9 |
| SPI-6 | Altered metabolic requirements | *S. enteritidis* (Sen) | AF376036 |
| NN^d^ | Iron uptake | *S. typhimurium* SL1344 (Sty) | AF128999 |
| CS54 island |  | *S. typhimurium* ATCC14028 (Sty) | AF140550(25.3) |
| SHI-1 | Enterotoxin, protease | *Shigella flexneri* 2a YSH6000T & SBA1336 (Sfl) | AF200692(51.3)^c^, U97487, U97489, U97491-2 |
| SHI-2 | Iron uptake | *S. flexneri* M90T & SA100 (Sfl) | AF141323(23.8)^c^, AF097520(14.3) |
| SRL | Iron uptake | *S. flexneri* 2a YSH6000 (Sfl) | AF326777(66.7) |
| SaPI1 | Toxic shock syndrome toxin 1, superantigen | *Staphylococcus aureus* RN4282 (Sau) | U93688(15.3)^c^, U93687 |
| SaPI3 | Enterotoxin | *S. aureus* COL (Sau) | AF410775(16.0)^c^ |
| SaPIbov | Toxic shock syndrome toxin 1, superantigen | *S. aureus* RF122(Sau) | AF217235(15.9)^c^ |
| SaPIbov2 | Biofilm formation | *S. aureus* V329 (Sau) | AY220730(27.0)^c^ |
| *Etd* PI | Exfoliative toxin, ETD | *S. aureus* TY114(Sau) | AB057421(14.8)^c^ |
| Yen HPI | Iron uptake | *Yersinia enterocolitica* Ye 8081 & WA314 (Yen) | X94452, X95298, AJ132668, AJ132945(14.0), Y12527(13.6) |
| Yps HPI | Iron uptake | *Y. pseudotuberculosis* PB1 & IP32637 (Yps); *Y. pestis* KIM10+ (Ype) | AJ236887, AJ009592, AJ009988 |
| VPI | Toxin-coregulated pilus (Tcp) adhesin, regulator | *Vibrio cholerae* 395; N16961; others (Vch) | AF325733(41.3)^c^, AF325734(41.3)^c^, AF034434(12.9), X64098(13.8), U39068(15.0), AF208385, AF319954, AF306795-8, AF319652-5, AF378526, AF452570-80 |
| CTX locus | Colera toxin (ctx) | *V. cholerae* N16961 & others (Vch) | U83796, AF220606, AF416590 |
| *cag* PAI | Type IV secretion, cytotoxing-associated gene (cag) antigen | *Helicobacter pylori* (Hpy) | AF282853(20.2)^c^, AF282852(21.3)^c^, U60177, AY136637-46 |
| *vap* region | Virulence-associated protein (Vap) antigens | *Dichelobacter nodosus* A198 (Dno) | L31763(12.8)^c^, X98545, X98547 |
| *Vrl* region | Virulence-related locus (Vrl) antigens | *D. nodosus* A198 (Dno) | U20246(28.1)^c^ |
| Hrp PAI | TTSS, effectors | *Pseudomonas syringae* DC3000 & others(Psy) | AF232004(52.5)^c^, AF232005(11.0), U25812-3, AF232003, AF069650-2, L41862, U03854-5, U07346, AF051694, L11582, AY147017-28 |
| Hrp PAI | TTSS, effectors | *Xanthomonas axonopodis* pv. glycines 8ra (Xax); *X. campestris* pv. vesicatoria 75-3 (Xca); *X. oryzae* pv. oryzae PXO86 & MAFF 311018 (Xor) | AF499777(29.2)^c^, U33548(16.9), AF056246(14.5), AF026197(10.1), AB045311(19.3), AB045312 |
| Hrp PAI | TTSS, effectors | *Burkholderia pseudomallei* E503 (Bps) | AF074878(29.8)^c^ |
| *hrp* locus^e^ | TTSS, effectors | *Ralstonia solanacearum* GMI1000 (Rso) | AJ245811(23.4) |
| Hrp PAI | TTSS, effectors | *Erwinia amylovora* Ea321 (Eam) | U56662 |
| PAGI-1 |  | *Pseudomonas aeruginosa* X24509 & PA14 (Pae) | AF241171(51.3)^c^, AY273869(111.3)^c^ |
| PAGI-2 |  | *P. aeruginosa* PA14 (Pae) | AY273870(13.8) |
| PaLoc |  | *Clostridium difficile* VPI10463 (Cdi) | X92982(26.0)^c^ |
| BfPAI | Fragilysin, metalloprotease II | *Bacteroides fragilis* VPI 13784 & 86-5443-2-2 (Bfr) | AF038459(6.8)^c^, AF118241-4 |
| NN^d^ |  | *Enterococcus faecalis* V583 (Efa) | AF454824(153.6)^c^ |
| *tc* locus | Toxin complex (tc) | *Photorhabdus luminescens* W14 (Plu) | AF346497(30.0), AF346498(16.2), AF346499(25.7) |
| TTSS locus | TTSS | *P. luminescens* W14 (Plu) | AY144116(47.7)^c^ |
| Mt locus | Macrophage toxin (Mt) | *P. luminescens* W14 (Plu) | AY144117(24.4) |

^a^Uropathogenic *E. coli* (UPEC), enteropathogenic *E. coli* (EPEC), enterohemorrhagic *E. col*i (EHEC), Shiga toxin-producing *E. coli* (STEC), rabbit-specific strains of enteropathogenic *E. coli* (REPEC)

^b^PAI loci of < 10kb are not listed.

^c^Fully sequenced PAI locus

^d^Unnamed PAI

^e^The *hrp* locus of *Ralstonia solanacearum* GMI1000 was not designated as a PAI in the strictest sense in the genome sequencing paper (Salanoubat et al. 2002. *Nature* 415:497-502)
