# Supplementary material for: A computational approach for identifying pathogenicity islands in prokaryotic genomes
Source: BMC Bioinformatics. 2005 Jul 21;6:184. doi: 10.1186/1471-2105-6-184 (PMC1188055; doi:10.1186/1471-2105-6-184)
Supplement: Additional File 3 — Detailed information of candidate PAIs in prokaryotic chromosomes [file 1471-2105-6-184-S3.doc]

Table 3S. Candidate PAIs in prokaryotic chromosomes

| **Strain (accession number)** | **Size (kb)** | **Position (bp)** | | **ΔG+C (%)^a^** | **HGT (%)^b^** | **Evidence of GI^c^** | **Characteristics** | **Homologs of virulence genes** | **PAI loci matched to this region (host strain^d^)** |
| --- | --- | --- | --- | --- | --- | --- | --- | --- | --- |
| *Bacillus halodurans* C-125^e^ (NC_002570) | 8.1 | 2,455,827 | -2.7 | | 100.0 | Transposase | ABC transporters | BH2335, BH2336 | PAI I_536_(UPEC) |
| *Bacillus subtilis* 168^e^ (NC_000964) | 4.9 | 1,704,258 | -2.3 | | 14.6 | - | Flagellar protein | *fliPQBA* | LEE(EPEC,EHEC,REPEC), SPI-2(Sty), Hrp(Bps), TTSS locus(Plu) |
| *Bordetella bronchiseptica* RB50 (NC_002927) | 15.3 | 1,723,214 | -1.6 | | 36.7 | tRNA | TTSS | *bcrH1H2, bscJNORSTUC, brpL* | LEE(EPEC,EHEC,REPEC,Cro), SPI-2(Sty), Hrp(Psy,Xax,Xca,Xor,Bps,Rso), TTSS locus(Plu) |
|  | 8 | 4,949,724 | 4.3 | | 93.2 | - | Hemin transport system | *hurIR*, *bhuUV* | SRL(Sfl) |
| *Bordetella pertussis* Tohama I (NC_002929) | 7.9 | 344,570 | 4.6 | | 93.1 | - | Heme uptake | *bhuVU*, *hurRI* | SRL(Sfl) |
|  | 15.3 | 2,363,093 | -1.2 | | 39.8 | tRNA | TTSS | *brpL*, *bscCUTSRONJ*, *bcrH2H1* | LEE(EPEC,EHEC,REPEC,Cro), SPI-2(Sty), Hrp(Psy,Xax,Xca,Xor,Bps,Rso), TTSS locus (Plu) |
| *Bradyrhizobium japonicum* USDA 110^e^ (NC_004463) | 6.4 | 1,968,299 | -4.3 | | 100.0 | - | Nodulation | *rhcNRSTU* | LEE(EPEC,EHEC,REPEC), SPI-2(Sty), Hrp(Bps,Rso), TTSS locus(Plu) |
| *Chromobacterium violaceum* ATCC 12472 (NC_005085) | 11.8 | 2,778,934 | -9.3 | | 100.0 | - | TTSS | *sseEC*, *cesD*, CV2280, 2283, *ssaGJL* | LEE(EHEC,REPEC,Cro), SPI-2(Sty) |
|  | 15.5 | 2,803,682 | -6.5 | | 86.4 | - | TTSS | *escCVRSTU*, CV2596,2599,2600,2603 | LEE(EPEC,EHEC,REPEC,Cro), SPI-2(Sty), Hrp(Psy,Xax,Xor,Bps,Rso), TTSS locus(Plu) |
| *Enterococcus faecalis* V583 (NC_004668) | 137.5 | 445,282 | -4.7 | | 83.8 | tRNA | **PAI in *E. faecalis*^f^** | *cylL-L*, *cylL-S*, *cylM*, EF0575-7, 0583-84 | PAI I_536_(UPEC), NN(Efa) |
| *Escherichia coli* CFT073 (NC_004431) | 7.1 | 370,224 | -6.3 | | 85.8 | tRNA, integrase, IS | Hypotheticals | c0392-3 | PAI III_536_(UPEC) |
|  | 60.1 | 1,180,751 | -1.8 | | 33.0 | tRNA, transposase, phage genes | F1C and S fimbrial protein, iron uptake | *mchF*, *sfaCBD*, *focACDFGHX*, *iroDCB*, c1256,1273 | PAI I~III_536_, IICFT073(UPEC), LEE(REPEC), SHI-1(Sfl), SRL(Sfl), NN(Sty) |
|  | 48.5 | 3,406,498 | -3.5 | | 46.1 | tRNA, integrase, transposase | **PAI I_CFT073_^f^** | c3557, *hlyCABD*, *papXGFEKJDCHAI*, c3610 | PAI I~III_536_, PAI I~II_CFT073_(UPEC), LEE(EHEC,STEC) |
|  | 29.1 | 3,480,253 | 2.7 | | 57.8 | IS | ISEc8, antigen 43 precursor, fimbrial protein | c3655 | PAI III_536_(UPEC) |
|  | 6 | 3,993,608 | -9.8 | | 87.1 | - | Fimbrial protein | c4208 | PAI I, III_536_(UPEC) |
|  | 43.3 | 4,910,097 | -2.7 | | 19.7 | Transposase | **PAI II_CFT073_^f^** | *cadAB*, c1574, *papGFEKJDCHAI* | PAI I~III_536_, PAI II_CFT073_(UPEC), LEE(REPEC), SHI-1(Sfl), SRL(Sfl) |
| *Escherichia coli* K12^e^ (NC_000913) | 9.8 | 557,435 | -3.8 | | 100.0 | Integrase, putative transposase | Fimbrial protein | *sfmACDF*, *fimZ* | PAI III_536_(UPEC) |
|  | 8.5 | 4,507,288 | 6.1 | | 43.3 | - | Iron transport | *fecEDCBRI* | SRL(Sfl) |
| *Escherichia coli* O157:H7 EDL933 (NC_002655) | 7 | 1,114,324 | -4.9 | | 42.2 | Putative transposase | Glucosyltransferase | Z1190 | PAI II,III_536_(UPEC) |
|  | 13.5 | 1,425,691 | -4.4 | | 100.0 | - | Pilin subunit, transporter and member of exoprotein | Z1534,1536-8, Z1543 | PAI II,III_536_(UPEC) |
|  | 7 | 1,509,932 | -4.9 | | 42.1 | Putative transposase, IS proteins | Glycosyl transferase, IS1 proteins | Z1629m | PAI II,III_536_(UPEC) |
|  | 14.9 | 3,786,504 | -13.3 | | 100.0 | tRNA | TTSS | Z4180,85,88,89,94,95,97 | LEE(EPEC,EHEC,REPEC,Cro), SPI-2(Sty), Hrp(Psy,Xor,Bps), TTSS locus(Plu) |
|  | 44.7 | 4,649,752 | -9.2 | | 87.3 | tRNA, integrase, phage genes | **LEE^f^** | *espABDF,escCDFJNRSTUV,sepLZQ,eae,Z5111,tir,Z5113,Z5125,cesD,Z5131,Z5140,Z5142* | PAI I~III_536_,II_CFT073_(UPEC), LEE(EPEC,EHEC,REPEC,Cro), SPI-2(Sty), SHI-1(Sfl), Hrp(Bps), TTSS locus(Plu) |
| *Escherichia coli* O157:H7 Sakai (NC_002695) | 7 | 1,426,158 | -4.6 | | 45.6 | Transposase | Ferric enterochelin esterase | ECs1370 | PAI II,III_536_(UPEC) |
|  | 17 | 3,717,102 | -14.0 | | 100.0 | tRNA | TTSS | ECs3712,3716,3718,3721,3724,3725,3730,3731,3733 | LEE(EPEC,EHEC,REPEC,Cro), SPI-2(Sty), Hrp(Psy,Xor,Bps), TTSS locus(Plu) |
|  | 44.7 | 4,580,769 | -9.4 | | 89.7 | tRNA | **LEE^f^** | *espFBDAG*, *escFDNVJUTSR*, *sepLQZD*, *intimin*, *cesTD*, *tir*, *ler*, ECs4562, 4579 | PAI I~III_536_, PAI II_CFT073_(UPEC), LEE(EPEC,EHEC,REPEC,Cro), SPI-2(Sty), SHI-1(Sfl), SRL(Sfl), Hrp(Bps), TTSS locus(Plu) |
| *Helicobacter pylori* 26695 (NC_000915) | 38 | 547,328 | -3.0 | | 82.6 | Glutamate racemase (*glr*) | ***cag* PAI^f^** | HP0520~0547 | Cag (Hpy) |
| *Helicobacter pylori* J99 (NC_000921) | 38.2 | 510,500 | -3.1 | | 83.7 | Glutamate racemase (*murI*) | ***cag* PAI^f^** | orf6,8,9,*virD4*,*virB11_1*,orf12~18,*cagTSQPMNLIHGFEDCA* | Cag (Hpy) |
| *Mesorhizobium loti* MAFF303099^e^ (NC_002678) | 12.7 | 5,155,372 | -5.5 | | 100.0 | - | TTSS, nodulation protein | *nolT*,mlr6342,8766,6345,6346,6348,msr8694 | LEE(EPEC,EHEC,REPEC,Cro), SPI-2(Sty), Hrp(Psy,Xor,Bps), TTSS locus(Plu) |
| *Nitrosomonas europaea* ATCC 19718^e^ (NC_004757) | 16.9 | 1,157,077 | 2.5 | | 34.9 | Recombinase | Transmembrane sensors, outer membrane efflux | NE1070-1,1078-9, 1085-6 | SRL(Sfl) |
| *Photorhabdus luminescens* subsp. laumondii TTO1 (NC_005126) | 23.2 | 888,552 | 8.3 | | 68.2 | - | Putative fimbrial proteins | *mrfABCDXEG,plu0780-0791* | PAI I~III_536_, PAI II_CFT073_(UPEC), PAI I (Eco AL862) |
|  | 36.3 | 1,227,925 | 7.7 | | 87.4 | tRNA, IS, transposase | Lipoprotein, pilus | *pilLNQRV* | PAGI-1(Pae) |
|  | 50.6 | 4,414,485 | -1.5 | | 17.0 | - | **TTSS locus^f^** | plu3751,53,55-60, *sctVYXWNOPQRSTUBCDFGHIJK*, plu64-5, plu3775-6 | LEE(EPEC,EHEC,REPEC,Cro), SPI-2(Sty), Hrp(Psy,Xor,Bps,Rso), TTSS locus(Plu) |
|  | 34.9 | 4,846,486 | 2.1 | | 64.1 | Transposase, IS | ***tc* locus^f^** | *tccC1B1A1* | *tc* locus (Plu) |
| *Salmonella enterica* Typhi Ty2 (NC_004631) | 6.7 | 24,473 | 0.6 | | 26.4 | - | Fimbrial protein | *bcfABEFG* | PAI II_CFT073_,III_536_ (UPEC) |
|  | 41.3 | 1,314,607 | -4.7 | | 61.2 | tRNA | SPI-2^f^ | *ttrACBSR*, *ssrBA*, *spiCA*, *ssaDEGHIJSTULVNOPQ*, *sseABCDEFG*, *sscAB*, t1279, *yscR* | LEE(EPEC,EHEC,REPEC,Cro), SPI-2(Sty), Hrp(Psy,Xax,Xor,Bps,Rso), TTSS locus(Plu) |
|  | 10.3 | 1,885,494 | -6.5 | | 57.7 | tRNA, transposase | SPI-5^f^ | *pipD*, *sigDE*, *pipBA* | SPI-5 (Sen) |
|  | 6.7 | 2,596,311 | -2.4 | | 95.5 | tRNA | Fimbrial protein | *stbABE* | PAI III_536_(UPEC), PAGI-1(Pae) |
|  | 12.4 | 2,868,755 | -5.2 | | 100.0 | - | SPI-1^g^ | *spaTSRQPI*,*invAG* | LEE(EPEC,EHEC,REPEC,Cro), SPI-2(Sty), Hrp(Psy,Xor,Bps,Rso), TTSS locus(Plu) |
|  | 25.5 | 4,306,060 | -7.3 | | 91.6 | - | SPI-4^f^ | t4162-4165,4168 | SPI-4(Sty) |
| *Salmonella enterica* Typhi CT18 (NC_003198) | 6.7 | 24,473 | 0.6 | | 26.4 | - | Fimbrial protein | *bcfABEFG* | PAI II_CFT073_,III_536_ (UPEC) |
|  | 6.7 | 378,099 | -2.5 | | 95.6 | tRNA | Fimbrial protein | *stbECBA* | PAI I,III_536_(UPEC), PAGI-1(Pae) |
|  | 10.3 | 1,085,068 | -6.6 | | 57.7 | tRNA, transposase | SPI-5^f^ | *pipABD*, *sigED* | SPI-5 (Sen) |
|  | 41.3 | 1,625,241 | -4.7 | | 61.2 | tRNA | SPI-2^f^ | *ssaUTSQPONVLJIHGED*, *yscR*, STY1709, *sseGFBA*, *sscBEDCA*, *spiAC*, *ssrAB*, *ttrRSBCA* | LEE(EPEC,EHEC,REPEC,Cro), SPI-2(Sty), Hrp(Psy,Xax,Xor,Bps,Rso), TTSS locus(Plu) |
|  | 12.4 | 2,882,898 | -5.2 | | 100.0 | IS, transposase | SPI-1^g^ | *spaTSRQPI*, *invAG* | LEE(EPEC,EHEC,REPEC,Cro), SPI-2(Sty), Hrp(Psy,Xor,Bps,Rso), TTSS locus(Plu) |
|  | 25.5 | 4,321,410 | -7.3 | | 91.6 | - | SPI-4^f^ | STY4452-4457,4460 | SPI-4(Sty) |
| *Salmonella typhimurium* LT2 (NC_003197) | 6.7 | 24,469 | 0.6 | | 26.4 | - | Fimbrial protein | *bcfABCEFG* | PAI I~III_536_, II_CFT073_(UPEC) |
|  | 8.3 | 376,663 | -3.5 | | 77.4 | - | Fimbrial protein | *stbECBA* | PAI I~III_536_(UPEC), PAGI-1(Pae) |
|  | 9.5 | 1,175,321 | -6.6 | | 52.2 | tRNA | **SPI-5^f^** | *pipABCD*, *sopB* | SPI-5(Sen) |
|  | 41.6 | 1,459,926 | -4.7 | | 60.9 | tRNA | **SPI-2^f^** | *ttrACBSR*, *ssrBA*, *ssaBCDEGHIJKLVNOPQRSTU*, *sseABCDEFG*, *sscAB* | LEE(EPEC,EHEC,REPEC,Cro), SPI-2(Sty), Hrp(Psy,Xax,Xor,Bps,Rso), TTSS locus(Plu) |
|  | 15.1 | 2,910,681 | 0.6 | | 100.0 | Putative transposase | Flagellar synthesis, siderophore receptor protein | *iroBCDEN* | PAI III_536_(UPEC) |
|  | 12.4 | 3,030,898 | -5.3 | | 100.0 | - | **SPI-1^g^** | *sicA*, *spaSRQP*, *invCAG* | LEE(EPEC,EHEC,REPEC,Cro), SPI-2(Sty), Hrp(Psy,Xor,Bps,Rso), TTSS locus(Plu) |
|  | 18 | 3,948,576 | -4.5 | | 56.8 | tRNA | **SPI-3^f^** | *misL*, *fidL*, *marT*, *slsA*, *cigR*, *mgtBC* | SPI-3 (Sty) |
|  | 25.5 | 4,476,286 | -7.5 | | 97.8 | - | **SPI-4^f^** | STM4257-4262 | SPI-4(Sty) |
| *Shigella flexneri* 2a 2457T (NC_004741) | 50.1 | 3,044,913 | -1.9 | | 17.2 | tRNA | **SHI-1^f^** | *sigA*, *pic* | PAI I~III_536_,II_CFT073_(UPEC), LEE(REPEC), SHI-1(Sfl), SRL(Sfl) |
|  | 25 | 3,943,536 | -2.3 | | 31.5 | tRNA | **SHI-2^f^** | *iutADCBA*, *imm*, *shiDCBA* | LEE(REPEC), SHI-2(Sfl), PAI II_CFT073_(UPEC) |
|  | 22.6 | 4,365,829 | -4.1 | | 48 | tRNA, recombinase | Fimbrial protein | *fimHGFCIA* | PAI I~II_536_,I~II_CFT073_(UPEC), LEE(EHEC,REPEC), SHI-2(Sfl), HPI(Yen), PAGI-2(Pae) |
| *Shigella flexneri* 2a 301 (NC_004337) | 13.7 | 507,845 | 1.8 | | 83.6 | Putative transposase | Enterochelin esterase, oxidoreductase (Fe-S subunit) | *fepA*, *fes* | PAI II_CFT073_,III_536_ (UPEC) |
|  | 7.5 | 1,731,146 | -3.1 | | 54.8 | tRNA | Oxidoreductases (Fe-S subunit) | *ydhXY* | SPI-2 (Sty) |
|  | 53.5 | 3,052,737 | -2.1 | | 8.1 | tRNA, integrase, transposase | **SHI-1^f^** | *sigA,pic,SF2976,sap* | PAI I~III_536_(UPEC), LEE(REPEC), SHI-1,2(Sfl), SRL(Sfl) |
|  | 28.1 | 3,806,770 | -2.5 | | 48.2 | tRNA, integrase, transposase | **SHI-2^f^** | *shiABCDF*, *iucABCD*, *iutA*, *lpfC* | SHI-2(Sfl), PAI IICFT073(UPEC) |
|  | 28.9 | 4,367,373 | -3.4 | | 50.1 | tRNA, integrase, transposase | Fimbrial protein | *fimHGFCIA* | PAI I~III_536_, PAI I,II_CFT073_(UPEC), LEE(EHEC,REPEC), SHI-2(Sfl), HPI(Yen), PAGI-2(Pae) |
| *Staphylococcus aureus* Mu50 (NC_002758) | 5.3 | 1,954,171 | -7.0 | | 100.0 | tRNA | SaPIm3^g^ | *seg*, *sen*, *sei*, *sem*, *seo* | SaPI1,3,bov (Sau) |
| *Staphylococcus aureus* MW2 (NC_003923) | 6.3 | 1,900,548 | 0.4 | | 43.8 | - | νSaβ^h^ | *splBA* | *etd* PI(Sau) |
| *Staphylococcus aureus* N315 (NC_002745) | 5.3 | 1,876,310 | -6.9 | | 100.0 | tRNA | SaPIn3^g^ | *seg*, *sen*, *sei*, *sem*, *seo* | SaPI1,3,bov (Sau) |
| *Vibrio cholerae* N16961 (NC_002505) | 42.9 | 873,242 | -11.5 | | 87.9 | Transposase | **VPI^f^** | VC0819~0845 | VPI (Vch) |
|  | 8.8 | 1,564,470 | -3.2 | | 100.0 | - | **CTX locus^h^** | *rstB1A1B2A2*, VC1455,1464 | CTX locus(Vch) |
| *Vibrio parahaemolyticus* RIMD 2210633 chromosome I (NC_004603) | 16.7 | 1,774,890 | 2.9 | | 39.6 | - | TTSS | VP1656~1675 | LEE(EPEC,EHEC,REPEC,Cro), SPI-2(Sty), Hrp(Psy,Xax,Xor,Bps,Rso), TTSS locus(Plu) |
|  | 11.3 | 1,801,133 | 0.0 | | 11.1 | - | TTSS, iron transport | VP1690~1701 | TTSS locus (Plu) |
| *Vibrio parahaemolyticus* RIMD 2210633 chromosome II (NC_004605) | 9 | 1,639,906 | 0.5 | | 26.1 | - | Flagellar biosynthesis | VPA1539,1542~1546 | Hrp(Psy,Bps), TTSS locus(Plu) |
|  | 3.7 | 1,767,656 | 4.9 | | 79.2 | - | Iron transport | VPA1652~1655 | SRL (Sfl) |
| *Xanthomonas campestris* pv. campestris ATCC 33913 (NC_003902) | 23.1 | 1,424,335 | -1.8 | | 10.0 | Transposase | **Hrp PAI^f^** | *hrpFED6D5B1B2B4B5B7FED6D5*, *hpaBAP12B*, *hrcSRQVUJNTC* | LEE(EPEC,EHEC,REPEC,Cro), SPI-2(Sty), Hrp(Psy,Xax,Xca,Xor,Bps,Rso), TTSS locus(Plu) |
| *Yersinia pestis* CO92 (NC_003143) | 34.7 | 2,142,992 | 9.1 | | 73.1 | tRNA, integrase | **HPI^f^** | *ybtETU*, irp1, *ybtAPQXS*, YPO1918,20,22 | PAI I,II_536_(UPEC), HPI(Yen,Yps), PAGI-1(Pae) |
|  | 8 | 2,738,739 | -1.7 | | 48.7 | Transposase | Iron transport system | *yfeABCD* | NN(Sty), NN(Efa) |
|  | 6.1 | 4,352,550 | -0.9 | | 100.0 | Transposase | Fimbrial protein, secreted protein | YPO3877-3879, 3881 | PAI I,III_536_(UPEC), PAGI-1(Pae) |
| *Yersinia pestis* KIM (NC_004088) | 34.7 | 2,639,085 | 9.1 | | 76.8 | tRNA, integrase | **HPI^f^** | *ybtSXQPATE*, y2388, 2390, 2392, *irp1* | PAI I,II_536_(UPEC), HPI(Yen) |
|  | 14.6 | 3,169,731 | -0.6 | | 5.6 | - | Iron transport, antigen chaperone | *yiuRC*,*psaCB* | PAI II_CFT073_(UPEC) |

^a^Deviation of the G+C content of the cPAI as compared to that of the whole genome

^b^Length percentage of horizontally transferred genes in the cPAI

^c^Genes involved in the transfer mechanism (integrase, transposase, IS element, or tRNA gene at the boundaries)

^d^Abbreviations of the strain names are denoted in supplementary Table 2S.

^e^Non-pathogenic bacterium

^f^cPAI that entirely matches to a PAI identified from the genome sequencing paper

^g^cPAI that matches to one end of a PAI identified from the genome sequencing paper. The other end of the PAI is present in a PAI-like region not overlapping GIs.

^h^cPAI that partly matches to a PAI identified from the genome sequencing paper

Bold characters denote that a sequenced strain containing the cPAI is the same as or closely related to the host strain of the queried PAI loci.
