# Supplementary material for: A computational approach for identifying pathogenicity islands in prokaryotic genomes
Source: BMC Bioinformatics. 2005 Jul 21;6:184. doi: 10.1186/1471-2105-6-184 (PMC1188055; doi:10.1186/1471-2105-6-184)
Supplement: Additional File 4 — Detailed information of PAI-like regions not overlapping GIs in prokaryotic chromosomes [file 1471-2105-6-184-S4.doc]

Table 4S. PAI-like regions not overlapping GIs in prokaryotic chromosomes

| **Strain (accession no.)** | **Size (kb)** | **Position (bp)** | **ΔG+C (%)^a^** | **HGT (%)^b^** | **Characteristics** | **PAI loci matched to this region (host strain^c^)** |
| --- | --- | --- | --- | --- | --- | --- |
| *Agrobacterium tumefaciens* C58 (Cereon) (NC_003063) | 3.5 | 461,374 | 0.1 | 0.0 | *sitABCD* | NN(Sty), NN(Efa) |
|  | 3.7 | 1,306,748 | 0.1 | 0.0 | Iron(III) uptake | SRL(Sfl) |
|  | 8.3 | 1,634,826 | 0.4 | 0.0 | Iron(III) uptake | SRL(Sfl) |
| *Agrobacterium tumefaciens* C58 (U. Washington) (NC_003305) | 8.3 | 431,662 | 0.4 | 0.0 | Iron(III) uptake | SRL(Sfl) |
|  | 3.7 | 764,298 | 0.1 | 0.0 | Iron uptake | SRL(Sfl) |
|  | 3.5 | 1,609,884 | 0.2 | 0.0 | Iron uptake | NN(Sty), NN(Efa) |
| *Bacillus anthracis* A2012 (NC_003995) | 5.0 | 2,098,520 | 0.8 | 0.0 | Flagellar biosynthesis | Hrp(Psy) |
| *Bacillus anthracis* Ames (NC_003997) | 5.0 | 1,610,765 | 0.4 | 0.0 | Flagellar biosynthesis | Hrp(Psy), TTSS locus(Plu) |
| *Bacillus halodurans* C-125 (NC_002570)^d^ | 4.9 | 2,560,176 | -0.3 | 0.0 | Flagellar biosynthesis | LEE(EPEC,UPEC,REPEC), Hrp(Psy,Bps), TTSS locus(Plu) |
|  | 4.8 | 3,415,831 | 0.1 | 0.0 | Iron uptake | SRL(Sfl) |
| *Bacillus subtilis* 168 (NC_000964)^d^ | 3.8 | 822,411 | 4.3 | 0.0 | Iron uptake | SRL(Sfl) |
| *Bordetella bronchiseptica* RB50 (NC_002927) | 7.8 | 2,741,150 | 1.8 | 0.0 | Flagellar biosynthesis | SPI-2(Sty) |
| *Bordetella parapertussis* 12822 (NC_002928) | 14.7 | 2,369,625 | -1.6 | 6.5 | TTSS | LEE(EPEC,UPEC,REPEC,Cro), SPI-2(Sty), Hrp(Psy,Xax,Xca,Xor,Bps,Rso), TTSS locus(Plu) |
| *Borrelia burgdorferi* B31 (NC_001318) | 5.1 | 283,869 | 1.8 | 0.0 | Flagellar biosynthesis | LEE(EPEC,UPEC,REPEC) |
| *Chromobacterium violaceum* ATCC 12472 (NC_005085) | 11.8 | 2,829,334 | 3.1 | 0.0 | Flagellar biosynthesis, surface presentation of antigens, invasion | LEE(EPEC,UPEC,REPEC,Cro), SPI-2(Sty), Hrp(Psy,Xor,Bps,Rso), TTSS locus(Plu) |
|  | 5.0 | 3,278,104 | 2.4 | 0.0 | Flagellar biosynthesis | Hrp(Psy), TTSS locus(Plu) |
| *Corynebacterium diphtheriae* NCTC13129 (NC_002935) | 4.7 | 141,608 | 1.7 | 0.0 | Membrane proteins, secreted protein | NN(Sty) |
| *Corynebacterium efficiens* YS-314 (NC_004369)^d^ | 10.3 | 727,298 | 3.4 | 0.0 | Iron uptake | SRL(Sfl) |
| *Escherichia coli* CFT073 (NC_004431) | 3.4 | 1,446,707 | -0.3 | 0.0 | *sitABCD* | NN(Sty), NN(Efa) |
|  | 9.4 | 1,476,306 | 1.7 | 2.0 | Hypotheticals | PAI I_CFT073_(UPEC) |
|  | 10.3 | 1,768,598 | -6.7 | 6.8 | Flagellar biosynthesis | PAI I,III_536_(UPEC) |
|  | 6.8 | 1,906,484 | -3.2 | 9.3 | Iron uptake | SPI-2(Sty) |
|  | 27.8 | 2,216,841 | 7.4 | 0.0 | ABC transporters, transmembrane protein, peptide/polyketide synthetase | HPI(Yen), PAI IV_536_(UPEC) |
|  | 9.3 | 3,461,193 | 2.6 | 0.0 | *lutADCB*, *shiF* | SHI-2(Sfl) |
|  | 8.2 | 5,137,789 | -0.8 | 0.0 | Fimbrial protein | PAI I~III_536_, PAI II_CFT073_(UPEC) |
| *Escherichia coli* K12 (NC_000913)^d^ | 6.8 | 997,082 | -3.4 | 8.1 | Fimbrial protein | PAI I,III 536 (UPEC), PAGI-1(Pae) |
|  | 6.8 | 1,748,369 | -3.4 | 9.3 | Oxidoreductase (Fe-S subunit) | SPI-2(Sty) |
|  | 9.0 | 2,066,974 | 3.4 | 5.0 | Outer membrane fluffing protein, DNA repair protein | PAI II,III_536_, II_CFT073_(UPEC), SHI-1, SRL(Sfl) |
|  | 8.2 | 4,540,683 | -1.0 | 0.0 | Fimbrial protein | PAI I~III_536_, PAI II_CFT073_(UPEC) |
| *Escherichia coli* O157:H7 EDL933 (NC_002655) | 5.7 | 650,325 | -4.7 | 10.2 | Fimbrial protein | PAI III_536_(UPEC) |
|  | 25.3 | 1,125,968 | 1.6 | 3.2 | Unknown in IS | PAI I~III_536_, PAI II_CFT073_(UPEC), LEE(REPEC), SHI-1, SRL(Sfl), HPI(Yen) |
|  | 6.8 | 1,216,554 | -3.0 | 8.1 | Fimbrial protein | PAI I,III_536_(UPEC), PAGI-1(Pae) |
|  | 20.1 | 1,521,574 | 1.4 | 4.1 | Unknowns in ISEc8 and IS629 | PAI I~III_536_, PAI II_CFT073_(UPEC), LEE(REPEC), SHI-1, SRL(Sfl) |
|  | 11.1 | 1,771,806 | 1.6 | 0.0 | Unknown in IS629 | PAI I_CFT073_(UPEC) |
|  | 10.3 | 1,974,046 | -6.1 | 1.9 | Fimbrial protein, oxidoreductase | PAI I,III_536_(UPEC) |
|  | 6.8 | 2,425,252 | -3.0 | 9.3 | Oxidoreductase (Fe-S subunit) | SPI-2(Sty) |
|  | 4.8 | 4,096,846 | -1.8 | 10.8 | Unknown in IS629 | PAI II_536_(UPEC) |
|  | 5.5 | 4,519,782 | -1.4 | 0.0 | Fimbrial protein | PAI III_536_(UPEC) |
|  | 8.1 | 5,428,009 | -0.7 | 0.0 | Fimbrial protein | PAI I~III_536_, PAI II_CFT073_(UPEC) |
| *Escherichia coli* O157:H7 Sakai (NC_002695) | 5.7 | 650,631 | -4.8 | 10.2 | Fimbrial protein | PAI III_536_(UPEC) |
|  | 6.1 | 1,127,331 | -3.7 | 9.0 | Fimbrial protein | PAI III_536_(UPEC) |
|  | 13.5 | 1,341,919 | -4.6 | 4.6 | Hemolysin-related | PAI II,III_536_(UPEC), PAGI-1(Pae) |
|  | 14.9 | 1,441,670 | 2.2 | 5.5 | DNA repair | PAI I~III_536_, PAI II_CFT073_(UPEC), LEE(REPEC), SHI-1, SRL(Sfl) |
|  | 11.1 | 1,680,399 | 1.5 | 0.0 | Hypotheticals | PAI I_CFT073_(UPEC) |
|  | 6.4 | 2,108,065 | -6.3 | 0 | Fimbrial protein | PAI III_536_(UPEC) |
|  | 6.8 | 2,350,056 | -3.2 | 9.3 | putative oxidoreductase (Fe-S subunit) | SPI-2(Sty) |
|  | 4.8 | 4,029,630 | -2.0 | 7.3 | Truncated putative Fimbrial protein | PAI II_536_(UPEC) |
|  | 8.1 | 5,398,006 | -0.9 | 0.0 | Fimbrial protein | PAI I~III_536_, PAI II_CFT073_(UPEC) |
| *Fusobacterium nucleatum* ATCC 25586 (NC_003454) | 3.5 | 1,324,109 | 0.2 | 0.0 | Zinc uptake system | PAI I_536_(UPEC), NN(Efa) |
| *Gloeobacter violaceus* PCC 7421 (NC_005125)^d^ | 4.7 | 3,508,438 | 1.4 | 16.2 | ABC transporters | PAI I_536_(UPEC), NN(Sty), NN(Efa) |
| *Haemophilus influenzae* Rd KW20 (NC_000907) | 6.6 | 380,643 | 1.9 | 11.1 | Iron uptake | PAI I_536_(UPEC), NN(Sty), NN(Efa) |
| *Halobacterium* sp. NRC-1 (NC_002607)^e^ | 7.1 | 1,912,908 | 1.3 | 0.0 | Iron uptake | SRL(Sfl) |
| *Listeria innocua* (NC_003212)^d^ | 5.0 | 710,716 | 2.0 | 0.0 | Flagellar biosynthesis | LEE(REPEC), Hrp(Psy) |
| *Listeria monocytogenes* EGD (NC_003210) | 4.9 | 209,470 | -0.8 | 6.6 | **LIPI-1^f^** | LIPI-1(Lmo) |
|  | 6.2 | 282,755 | -1.6 | 0.0 | **Internalin^f^** | Internalin(Lmo) |
|  | 5.0 | 711,428 | 2.4 | 0.0 | Flagellar biosynthesis | LEE(EPEC), Hrp(Psy) |
|  | 3.6 | 1,859,272 | -1.7 | 0.0 | **Internalin^f^** | Internalin(Lmo) |
|  | 5.8 | 2,029,698 | 1.5 | 0.0 | Iron uptake | SRL(Sfl) |
| *Nitrosomonas europaea* ATCC 19718 (NC_004757)^d^ | 9.5 | 178,919 | 2.7 | 3.2 | ABC transporter, secretion protein | PAI II_536_(UPEC) |
|  | 1.9 | 519,527 | -1.3 | 0.0 | Hypotheticals | Vap region(Dno) |
|  | 8.0 | 1,187,647 | 2.6 | 3.4 | Hypotheticals | SRL(Sfl) |
| *Nostoc* sp. PCC 7120 (NC_003272)^d^ | 13.2 | 456,014 | 1.7 | 0.0 | Hypotheticals | SHI-2(Sfl) |
|  | 11.4 | 2,650,904 | -2.6 | 0.0 | Iron uptake | SRL(Sfl) |
|  | 4.1 | 3,088,246 | 0.6 | 0.0 | Iron uptake | SRL(Sfl) |
| *Oceanobacillus iheyensis* HTE831 (NC_004193)^d^ | 8.6 | 452,539 | 1.6 | 0.0 | ABC transporters, transcriptional regulator | PAI I_536_(UPEC), NN(Efa) |
|  | 7.2 | 473,658 | 0.6 | 0.0 | Iron uptake | SRL(Sfl) |
|  | 4.9 | 1,609,891 | -0.9 | 0.0 | Flagellar biosynthesis | LEE(EPEC,EHEC,REPEC,Cro), SPI-2(Sty), Hrp(Psy,Bps,Rso), TTSS locus(Plu) |
| *Pasteurella multocida* PM70 (NC_002663) | 6.9 | 148,935 | 1.0 | 0.0 | Iron uptake | SRL(Sfl) |
|  | 3.5 | 465,768 | -0.8 | 0.0 | Unkowns | NN(Sty,Efa) |
|  | 7.3 | 849,141 | 1.4 | 0.0 | *ttrACBSR* | SPI-2(Sty) |
| *Photorhabdus luminescens* subsp. laumondii TTO1 (NC_005126) | 7.3 | 278,598 | 5.0 | 0.0 | Hypotheticals | PAI I,III_536_(UPEC), NN(Eco862) |
|  | 5.6 | 442,088 | 5.4 | 0.0 | Hypotheticals | PAI II_536_, II_CFT073_(UPEC) |
|  | 23.3 | 2,716,778 | 4.8 | 0.0 | Hypotheticals | HPI(Yen) |
|  | 3.6 | 3,175,519 | 0.0 | 0.0 | Iron uptake | NN(Sty), NN(Efa) |
|  | 7.6 | 5,193,038 | 9.6 | 20.0 | Iron uptake | SRL(Sfl) |
| *Pseudomonas aeruginosa* PA01 (NC_002516) | 8.0 | 1,574,475 | 0.2 | 0.0 | Flagellar biosynthesis | Hrp(Psy), TTSS locus(Plu) |
|  | 25.7 | 1,840,468 | 0.3 | 4.3 | TTSS | LEE(EPEC,EHEC,REPEC,Cro), SPI-2(Sty), Hrp(Psy,Xax,Xor,Bps,Rso), TTSS locus(Plu) |
|  | 9.2 | 2,339,352 | 0.8 | 0.0 | Fimbrial protein | PAI I,III_536_(UPEC), PAGI-1(Pae) |
|  | 10.6 | 4,559,005 | -1.4 | 13.1 | Fimbrial protein, pilin | PAI I_536_(UPEC), PAGI-1(Pae) |
|  | 17.9 | 4,726,892 | 5.5 | 0 | ATP transporter, pyochelin biosynthesis | HPI (Yen) |
| *Pseudomonas putida* KT2440 (NC_002947)^d^ | 12.9 | 5,604,523 | 0.8 | 0.0 | Lipopolysaccharide biosynthesis, efflux transporters | PAI II_536_(UPEC) |
| *Pseudomonas syringae* pv. tomato DC3000 (NC_004578) | 8.1 | 803,869 | 3.9 | 0.0 | Iron uptake, oxidoreductase | SRL(Sfl) |
|  | 9.3 | 1,316,530 | 1.8 | 0.0 | Iron uptake | SRL(Sfl) |
|  | 50.6 | 1,502,395 | -0.9 | 3.6 | **Hrp PAI^g^** | LEE(EPEC,EHEC,REPEC,Cro), SPI-2(Sty), Hrp(Psy,Eam,Psy), TTSS locus(Plu) |
|  | 5.5 | 2,157,121 | 0.0 | 0.0 | Flagellar biosynthesis | Hrp(Psy,Xor,Bps), TTSS locus(Plu) |
|  | 23.9 | 2,870,283 | 5.5 | 0.0 | Iron uptake | HPI(Yen) |
|  | 13.6 | 6,369,107 | -2.6 | 0 | Unkowns in IS | LEE(EHEC) |
| *Salmonella enterica* Typhi Ty2 (NC_004631) | 17.1 | 389,798 | 5.1 | 0.0 | Exported proteins | CS54 island(Sty) |
|  | 6.1 | 2,378,933 | 1.9 | 0.0 | Fimbrial protein | PAI I,III_536_(UPEC) |
|  | 9.4 | 2,755,306 | 4.3 | 0.0 | Iron uptake | PAI III_536_(UPEC) |
|  | 3.4 | 2,845,119 | 1.5 | 0.0 | SPI-1^h^ | NN(Sty), NN(Efa) |
|  | 8.1 | 3,077,594 | 1.5 | 5.7 | ABC-transporters | SRL(Sfl) |
|  | 18.3 | 3,867,695 | -4.5 | 7.8 | SPI-3^g^ | SPI-3(Sty) |
|  | 15.5 | 4,394,302 | -1.4 | 7.9 | Hypotheticals | PAGI-1(Pae) |
|  | 24.4 | 4,418,334 | -2.7 | 5.5 | Hypotheticals | PAGI-1(Pae) |
| *Salmonella enterica* Typhi CT18 (Salmonella enterica Typhi Typhi) (NC_003198) | 6.1 | 596,077 | 1.8 | 0.0 | Fimbrial protein | PAI I,III_536_(UPEC) |
|  | 17.1 | 2,597,945 | 5.0 | 0.0 | Exported proteins | CS54 island(Sty) |
|  | 9.4 | 2,769,449 | 4.2 | 0.0 | Iron uptake | PAI III536(UPEC) |
|  | 3.4 | 2,859,262 | 1.4 | 0.0 | SPI-1^h^ | NN(Sty), NN(Efa) |
|  | 8.1 | 3,092,099 | 1.4 | 5.7 | ABC-transporters | SRL(Sfl) |
|  | 18.3 | 3,882,205 | -4.6 | 7.8 | SPI-3^g^ | SPI-3(Sty) |
|  | 15.5 | 4,409,652 | -1.4 | 7.9 | Hypotheticals | PAGI-1(Pae) |
| *Salmonella typhimurium* LT2 (NC_003197) | 10.3 | 225,976 | 2.0 | 4.6 | Iron uptake | PAI II,III_536_(UPEC) |
|  | 6.1 | 604,115 | 1.7 | 0.0 | Fimbrial protein | PAI I~III_536_(UPEC) |
|  | 23.2 | 2,627,589 | 4.8 | 0.0 | Membrane proteins | CS54 island(Sty) |
|  | 3.4 | 3,006,368 | 1.3 | 0.0 | SPI-1^h^ | NN(Sty), NN(Efa) |
|  | 5.2 | 3,275,333 | -4.7 | 8.4 | Transcriptional regulators, putatives | SPI-6(Sen) |
|  | 5.5 | 3,822,485 | -1.5 | 0.0 | Fimbrial protein | PAI III_536_(UPEC) |
|  | 4.5 | 4,848,937 | -4.6 | 12.5 | Fimbrial protein | PAI III_536_(UPEC) |
| *Shewanella oneidensis* MR-1 (NC_004347)^d^ | 5.5 | 3,353,135 | 0.0 | 0.0 | Flagellar biosynthesis | TTSS locus(Plu) |
| *Shigella flexneri* 2a 2457T (NC_004741) | 5.9 | 989,833 | -2.4 | 4.7 | Unknown in IS1, fimbrial protein | PAI III_536_(UPEC) |
|  | 11.6 | 1,225,471 | 0.5 | 0.0 | Hypotheticals | PAI I_CFT073_(UPEC) |
|  | 7.5 | 1,769,442 | -3.1 | 12.0 | Unknowns in IS1, oxidoreductase (Fe-S subunit) | SPI-2(Sty) |
|  | 19.7 | 1,892,281 | -1.3 | 5.3 | Iron uptake, unknowns in IS1, IS2 IS629, and IS911, bacteriophage related proteins | PAI I_536_, PAI II_CFT073_(UPEC), NN(Sty), SHI-2(Sfl), NN(Efa) |
|  | 2.7 | 4,454,885 | -1.4 | 20.5 | Unkowns in IS1 and IS600 | PAI III_536_(UPEC) |
| *Shigella flexneri* 2a 301 (NC_004337) | 7.1 | 984,651 | -1.6 | 7.8 | Fimbrial protein | PAI III_536_(UPEC) |
|  | 13.4 | 1,222,570 | 0.4 | 0.0 | Hypotheticals | PAI I_CFT073_(UPEC) |
|  | 25.8 | 1,401,173 | -2.0 | 3.8 | Iron uptake, invaseion plamid antigen | PAI I_536_, II_CFT073_(UPEC), NN(Sty), SHI-2(Sfl), NN(Efa) |
|  | 7.6 | 1,568,246 | -4.8 | 7.3 | Hypotheticals | PAI III_536_(UPEC) |
| *Sinorhizobium meliloti* (NC_003047)^d^ | 3.5 | 3,283,929 | 0.5 | 0.0 | Iron uptake | NN(Sty), NN(Efa) |
| *Staphylococcus aureus* Mu50 (NC_002758) | 7.2 | 1,935,792 | 0.0 | 0.0 | SaPIm3^h^ | *etd* PI(Sau) |
|  | 15.1 | 2,133,661 | -2.3 | 8.3 | SaPIm1^g^ | SaPI1,3,bov,bov2(Sau) |
| *Staphylococcus aureus* MW2 (NC_003923) | 11.5 | 841,945 | -4.4 | 7.9 | νSa3^g^ | SaPI1,3(Sau) |
| *Staphylococcus aureus* N315 (NC_002745) | 7.2 | 1,857,930 | 0.0 | 0.0 | SaPIn3^h^ | *etd* PI(Sau) |
|  | 15.1 | 2,057,159 | -2.3 | 11.7 | SaPIn1^g^ | SaPI1,3,bov,bov2(Sau) |
| *Streptococcus agalactiae* 2603V/R (NC_004116) | 8.8 | 1,539,918 | -0.1 | 0.0 | Hypotheticals | PAI I_536_(UPEC) |
| *Streptococcus agalactiae* NEM316 (NC_004368) | 5.3 | 1,239,217 | -1.7 | 0.0 | Hypotheticals | NN(Efa) |
| *Streptococcus pneumonia* R6 (NC_003098) | 7.9 | 292,900 | 0.0 | 3.1 | PTS(Phosphotransferase system) | NN(Efa) |
|  | 6.7 | 1,469,087 | 2.7 | 0.0 | PTS | NN(Sty) |
| *Streptococcus pneumonia* TIGR4 (NC_003028) | 10.2 | 295,247 | 0.2 | 0.0 | PTS | NN(Efa) |
| *Streptococcus pyogenes* SSI-1 (NC_004606) | 9.3 | 918,855 | 3.7 | 11.9 | PTS | NN(Efa) |
| *Streptomyces coelicolor* A3(2) (NC_003888)^d^ | 25.1 | 8,504,461 | 2.1 | 0.0 | ABC transporters | HPI(Yen) |
| *Synechococcus* sp. WH8102 (NC_005070)^d^ | 5.8 | 1,327,616 | 3.6 | 0.0 | Iron uptake | NN(Sty) |
| *Synechocystis* sp. PCC6803 (NC_000911)^d^ | 4.2 | 13,498 | 2.8 | 0.0 | Iron uptake | SRL(Sfl) |
|  | 9.1 | 947,511 | 2.3 | 0.0 | ABC transporter | PAI II_536_(UPEC) |
| *Thermoanaerobacter tengcongensis* MB4T (NC_003869)^d^ | 11.7 | 379,422 | -0.5 | 0.0 | ABC-type Mn/Zn transport systems | NN(Sty) |
|  | 4.9 | 1,401,516 | 1.7 | 0.0 | TTSS, Flagellar biosynthesis | LEE(EPEC,EHEC,REPEC,Cro), SPI-2(Sty), Hrp(Psy,Xor,Bps), TTSS locus(Plu) |
| *Treponema pallidum* (NC_000919) | 3.9 | 184,611 | 0.0 | 0.0 | ABC transporters | PAI I_536_(UPEC) |
|  | 5.2 | 781,513 | -8.8 | 15.4 | Flagellar biosynthesis | TTSS locus(Plu) |
| *Vibrio parahaemolyticus* RIMD 2210633 (NC_004603) | 7.7 | 2,103,438 | 4.4 | 12 | Hypothetical | SPI-2(Sty) |
| *Vibrio vulnificus* CMCP6 (NC_004459) | 5.4 | 1,935,569 | 2.7 | 0.0 | Flagellar biosynthesis | Hrp(Psy) |
| *Vibrio vulnificus* YJ016 (NC_005139) | 7.6 | 2,237,581 | 3.5 | 0.0 | Hypotheticals | SPI-2(Sty) |
|  | 5.4 | 2,500,964 | 2.7 | 0.0 | Flagellar biosynthesis | Hrp(Psy) |
| *Xanthomonas axonopodis* pv. citri 306 (NC_003919) | 27.6 | 462,225 | -2.7 | 4.2 | Hypotheticals | LEE(EPEC,EHEC,REPEC,Cro), SPI-2(Sty), Hrp(Psy,Xax,Xca,Xor,Bps,Rso), TTSS locus(Plu) |
|  | 14.9 | 2,262,166 | 2.0 | 4.0 | Flagellar biosynthesis | Hrp(Psy,Rso), TTSS locus(Plu) |
| *Xanthomonas campestris* pv. campestris ATCC 33913 (NC_003902) | 15.0 | 2,234,279 | 2.6 | 0.0 | Flagellar biosynthesis | Hrp(Psy,Rso), TTSS locus(Plu) |
| *Yersinia pestis* CO92 (NC_003143) | 18.4 | 255,039 | 1.5 | 8.3 | TTSS | LEE(EPEC,EHEC,REPEC,Cro), SPI-2(Sty), Hrp(Psy,Xax,Xor,Bps,Rso), TTSS locus(Plu) |
|  | 9.0 | 770,790 | 0.7 | 0.0 | Flagellar biosynthesis | Hrp(Psy) |
|  | 6.7 | 835,033 | -0.8 | 0.0 | ABC transporters | HPI(Yen) |
|  | 12.7 | 1,096,175 | 3.2 | 0.0 | Iron uptake, IS1661 related protein | SHI-2(Sfl) |
|  | 14.6 | 1,463,379 | -0.6 | 0.0 | Iron uptake, outer membrane proteins, endonuclease IV | PAI IICFT073(UPEC) |
|  | 6.1 | 1,948,363 | -1.0 | 8.9 | Fimbrial protein | PAI I_536_(UPEC), PAGI-1(Pae) |
|  | 7.2 | 2,066,410 | 4.8 | 5.1 | Flagellar biosynthesis | TTSS locus(Plu) |
|  | 3.8 | 2,183,470 | 3.3 | 0.0 | Transcriptional regulators | SPI-6(Sen) |
| *Yersinia pestis* KIM (NC_004088) | 6.1 | 386,990 | -0.9 | 8.6 | Fimbrial protein | PAI I,III_536_(UPEC), PAGI-1(Pae) |
|  | 18.4 | 581,872 | 1.5 | 7.8 | TTSS | LEE(EPEC,EHEC,REPEC,Cro), SPI-2(Sty), Hrp(Psy,Xax,Xor,Bps,Rso), TTSS locus(Plu) |
|  | 6.1 | 2,063,668 | -1.0 | 8.9 | Fimbrial protein | PAI I_536_(UPEC), PAGI-1(Pae) |
|  | 8.0 | 2,087,489 | -1.7 | 2.7 | Iron and manganese ABC transporters | NN(Sty), NN(Efa) |
|  | 3.8 | 2,629,576 | 3.3 | 0.0 | Transcriptional regulators | SPI-6(Sen) |
|  | 7.3 | 2,743,187 | 4.9 | 7.4 | Flagellar biosynthesis | TTSS locus(Plu) |
|  | 12.7 | 3,724,277 | 3.2 | 0.0 | Iron uptake (aerobactin OM receptor, aerobactin synthetases) | SHI-2(Sfl) |
|  | 6.7 | 3,791,123 | -0.8 | 0.0 | Hypotheticals | HPI(Yen) |
|  | 9.0 | 3,843,421 | 0.7 | 0.0 | Flagellar biosynthesis | Hrp(Psy) |
|  | 7.6 | 4,504,671 | -4.4 | 0.0 | Fimbrial protein | PAI II_CFT073_(UPEC) |

^a^Deviation of the G+C content of the region as compared to that of the whole genome.

^b^Length percentage of horizontally transferred genes in the region

^c^Abbreviations of strain names are denoted in supplementary Table 2S.

^d^Non-pathogenic bacterium

^e^Archeaon

^f^Region that partly matches to a PAI identified from the genome sequencing project

^g^Region that entirely matches to PAI identified from the genome sequencing paper.

^h^Region that matches to one end of a PAI identified from genome sequencing paper. The other end of each PAI is present in a cPAI.

Bold characters denote that a sequenced strain containing the PAI-like region is the same as or closely related to the host strain of the queried PAI loci. The entire region of the *hrp* locus in *Ralstonia solanacearum* GMI1000 was detected in the megaplasmid pGMI1000MP (NC_003296).
